# Phosphorylation of the histone demethylase KDM5B and regulation of the phenotype of triple negative breast cancer

**DOI:** 10.1038/s41598-019-54184-0

**Published:** 2019-11-27

**Authors:** I-Ju Yeh, Emily Esakov, Justin D. Lathia, Masaru Miyagi, Ofer Reizes, Monica M. Montano

**Affiliations:** 1 Department of Pharmacology, Case Western Reserve University Cleveland, Cleveland, OH 44106 USA; 2 Department of Cellular and Molecular Medicine, Cleveland Clinic Lerner Research Institute, 9500 Euclid Ave., Cleveland, OH 44195 USA

**Keywords:** Breast cancer, Breast cancer

## Abstract

Epigenetic modifications are known to play critical roles in the expression of genes related to differentiation and dedifferentiation. Histone lysine demethylase KDM5B (PLU-1) catalyzes the demethylation of histone H3 on Lys 4 (H3K4), which results in the repression of gene expression. KDM5B is involved in regulation of luminal and basal cell specific gene expression in breast cancers. However, the mechanisms by which KDM5B is regulated in breast cancer, in particular in response to post-translational signals is not well-defined. Here, we demonstrate that KDM5B is phosphorylated at Ser1456 by the cyclin-dependent kinase 1 (CDK1). Phosphorylation of KDM5B at Ser1456 attenuated the occupancy of KDM5B on the promoters of pluripotency genes. Moreover, KDM5B inhibited the expression of pluripotency genes, *SOX2* and *NANOG*, and decreased the stem cell population in triple-negative breast cancer cell lines (TNBC). We previously reported that the tumor suppressor HEXIM1 is a mediator of KDM5B recruitment to its target genes, and HEXIM1 is required for the inhibition of nuclear hormone receptor activity by KDM5B. Similarly, HEXIM1 is required for regulation of pluripotency genes by KDM5B.

## Introduction

Triple Negative Breast Cancer (TNBC) is a breast cancer subtype that does not express the estrogen receptor (ER), progesterone receptor (PR) and human epidermal growth factor receptor-2 (HER2)^1^. Clinically, 70% of TNBC tumors belong to the basal subtype, which are more aggressive, less differentiated, and have enhanced metastatic capacity^2^. Although TNBC tumors have been reported to be more responsive to neoadjuvant chemotherapy than receptor-positive tumors, development of resistance to cytotoxic chemotherapy and early relapse are more common^3^. Due to the lack of effective therapies resulting in poor prognosis for patients with TNBC, understanding the underlying mechanisms of TNBC aggressiveness is of key importance and thus a major focus of research in the hopes that new therapeutic targets will be elucidated.

Cancer stem cells (CSCs) are a population of cells within tumors that exhibit multipotency, self-renewal, clonogenicity, and proliferative properties^4^. CSCs can be sorted by a unique phenotype of surface proteins such as CD44^+^CD24^-^ and characterized by increased aldehyde dehydrogenase (ALDH) activity^5,6^. Published reports indicate that CSCs contribute to therapeutic-resistance and metastasis of malignant breast cancer cells^7,8^. Furthermore, biopsy samples from cancer patients that have higher percentages of CSCs are more likely to metastasize^9^. The CD44^+^/CD24^−^/ALDH^+^ population is enriched in certain TNBC subtypes^6,10,11^, and may contribute to the aforementioned phenotype of TNBC tumors.

Epigenetic mechanisms are key factors in the emergence of CSCs^12^. At the initial stage of cell fate determination, maintaining the balance of H3K4 methylation is essential^13,14^, and the choice between differentiation versus dedifferentiation partly depends on this dynamic balance of methylation markers. Epigenetic enzymes, such as KDM5B, play essential roles in this decision. Along this line, KDM5B, a demethylase of histone 3 methylated on lysine 4, directly regulates genes that control cell cycle, cell differentiation, and cell lineage in murine embryonic stem cells (mECs) during the early epiblast, thus influencing cell fate decisions^15^.

High expression of KDM5B in prostate and breast cancer cells supports a proliferative role of KDM5B^16–18^. shRNA knockdown of KDM5B inhibits proliferation of several cancer cell lines and xenograft models^19–21^. However, a tumor-suppressive role of KDM5B has also been reported in TNBC, partly due to its ability to bind and stabilize hypophosphorylated pRb^22–24^. We previously reported that a functional interaction between KDM5B and the tumor suppressor, HEXIM1, is required for HEXIM1 inhibition of nuclear hormone receptor activity^25^. These divergent roles of KDM5B suggest that the role of KDM5B is cell-type dependent and dictated by KDM5B interacting partners.

Cyclin-dependent kinase 1 (CDK1) is a critical regulator of the cell cycle and its activity is highest during G_2_/M transition. CDK1 inhibition resulted in attenuated growth of TNBC tumor xenografts that have elevated Myc expression by inducing the apoptotic response^26^. Paradoxically, high expression of CDK1 in clinical invasive breast cancer is associated with better DRFSC (Distant relapse-free survival)^27^, suggesting the CDK1 may also have divergent roles in cancer.

CDK1 has been reported to be a critical factor in maintaining pluripotency of human stem cells^28^. In this report, we show that CDK1 phosphorylated KDM5B and in doing so downregulated KDM5B recruitment to genes encoding *SOX2* and *NANOG*. Downregulation of KDM5B expression resulted in increased Sox2 and Nanog expression, and increase in CSC population. However, the role of CDK1 is more complex, and reflect CDK1 regulation of the recruitment of another histone demethylase, KDM4B.

## Results

### Phosphorylation of KDM5B by CDK1

**Figure 1:**
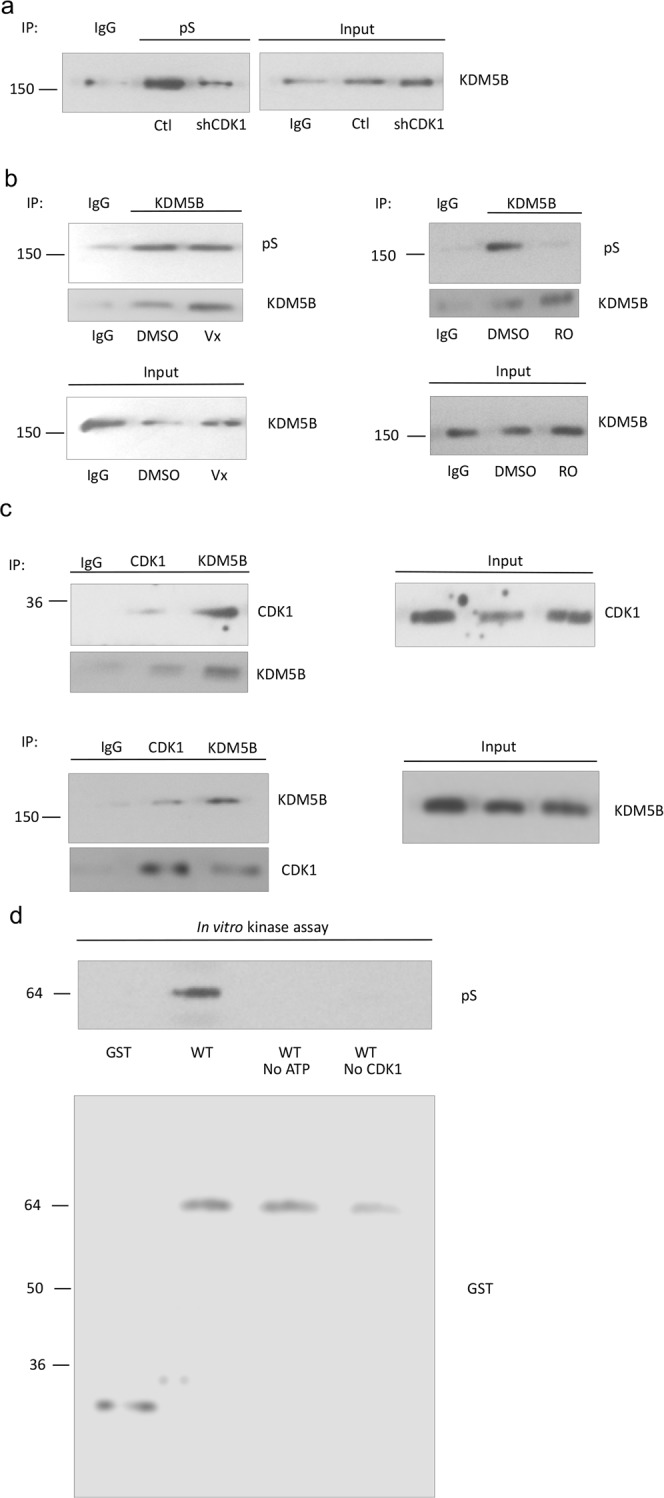
KDM5B is serine phosphorylated by CDK1. MDA-MB-231 cells were infected with control shRNA lentiviruses or CDK1 shRNA lentiviruses. (**a**) Lysates from MDA-MB-231 cells were immunoprecipitated using antibodies against phosphorylated serine (pS) and analyzed for KDM5B by Western blotting. (**b**) MDA-MB-231 cells were treated with kinase inhibitors (Erk inhibitor, Vx: Vx-11e, 100 μM or CDK1 inhibitor, RO: RO3306, 10 μM) and lysates were immunoprecipitated using antibodies against KDM5B and analyzed for phosphorylated serine by Western blotting. (**c**) Lysates from MDA-MB-231 cells were immunoprecipitated using antibodies against KDM5B and CDK1 and analyzed for co-immunoprecipitation of CDK1 and KDM5B, respectively, by Western blotting. Normal rabbit IgG was used as a negative control. Input lanes represent 25% of the total protein. (**d**) Upper panel: *In vitro* kinase assay wherein recombinant cyclin B1 were incubated with purified GST-KDM5B in the absence or presence of CDK1 or ATP. Phosphoserine signal was detected by Western blotting. Lower panel: Western blot analyses of purified GST-KDM5B used in kinase assays. Figures are representative of at least 3 independent experiments.

KDM5B, a H3K4me2/3 histone demethylase, has been reported to be overexpressed in human breast tumors and proposed to have an oncogenic role^21,29,30^. As a histone demethylase, KDM5B is involved in the transcriptional regulation of several genes, in particular those involved in differentiation and dedifferentiation^30–32^. Although phosphorylation has been reported to play roles in the regulation of other histone demethylases, regulation of KDM5B activity via phosphorylation has not been reported. Our immunoprecipitation experiments supported serine phosphorylation of KDM5B in a triple negative breast cancer cell line, MDA-MB-231 (Fig. 1a). Potential KDM5B phosphorylation sites were determined using KinasePhos. The CDK1 phosphorylation site was of specific interest due to its high score, and CDK1 was therefore explored for its involvement in the regulation of KDM5B phosphorylation. Serine phosphorylation of KDM5B was decreased in cells transfected with CDK1 shRNA when compared to control shRNA (Fig. 1a). Moreover, the phosphorylation of KDM5B was attenuated in RO3306-treated cells, a selective CDK1 inhibitor (Fig. 1b). Further support for phosphorylation of KDM5B by CDK1 is the interaction between CDK1 and KDM5B based on co-immunoprecipitation of endogenous CDK1 and KDM5B (Fig. 1c). *In vitro* CDK1 kinase assays were utilized to demonstrate that CDK1 catalyzed the phosphorylation of KDM5B, but not glutathione S-transferase (GST) alone (Fig. 1d).

### Identification of CDK1 phosphorylation sites

To identify residues phosphorylated by CDK1 we used both mass spectrometry as well as in silico/predictive approaches. We used both approaches due to limitations of mass spectrometry^33^ and reports of functionally relevant phosphorylation sites not detected by mass spectrometry^34–38^.

**Figure 2:**
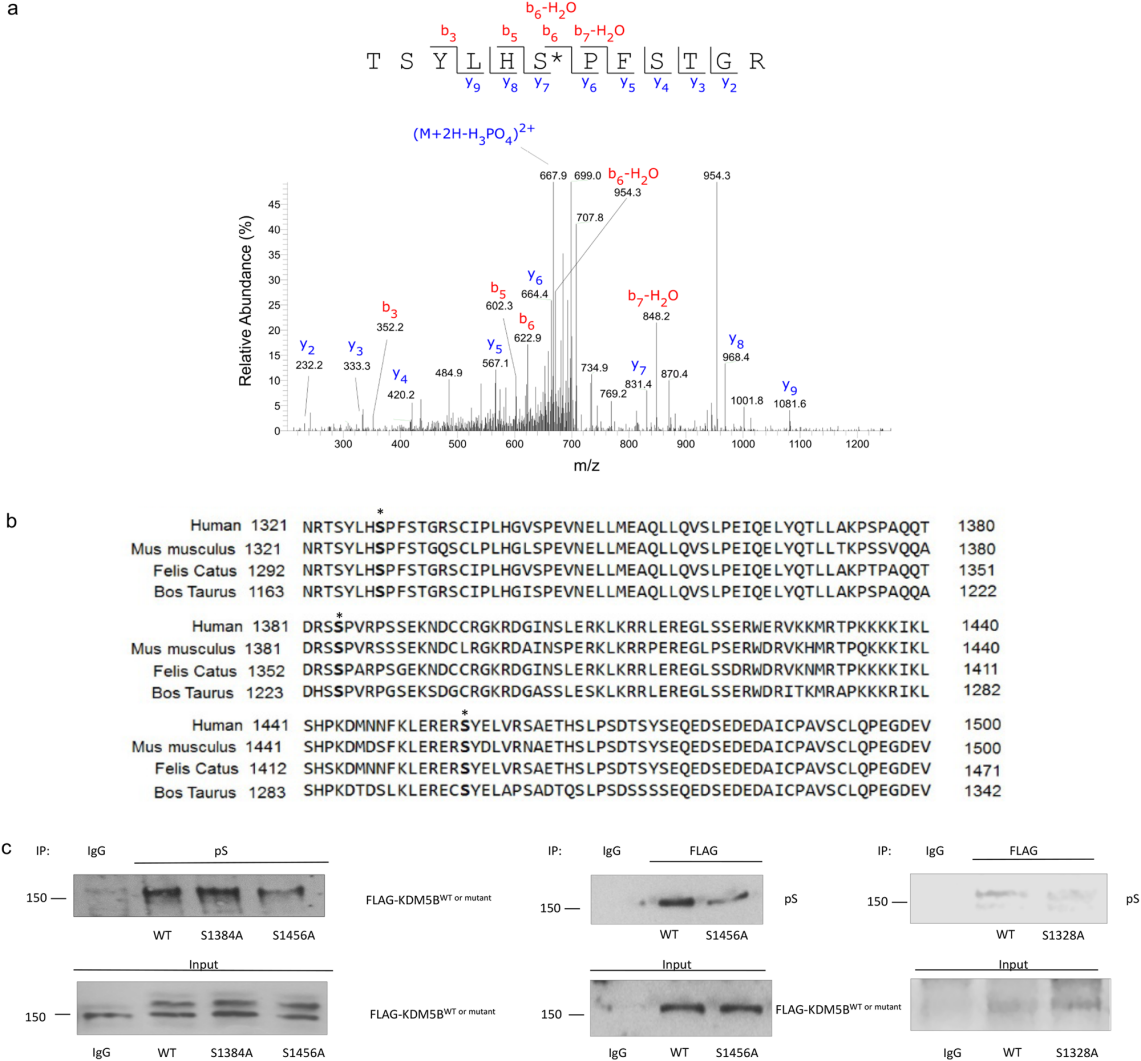
KDM5B is phosphorylated at S1456 and S1328. (**a**) Cyclin B, CDK1 and GST–KDM5B (1156–1544) were subjected to an *in vitro* kinase assay and analyzed by mass spectrometry. Shown is tandem mass spectra of phosphorylated peptides from KDM5B. Observed b- and y-series ions are shown in each spectrum. MS/MS spectrum of a peptide containing phospho-Ser1328 (precursor ion: m/z 716.8, +2 charge). (**b**) PRABI sequence alignment of orthologous KDM5B C-terminal region. MDA-MB-231 cells were transfected with expression vectors for FLAG-KDM5B^WT^, FLAG-KDM5B^S1384A^, FLAG-KDM5B^S1456A^, or FLAG-KDM5B^S1328A^. (**c**) Left panels: Lysates were immunoprecipitated using antibodies against phosphorylated serine. FLAG-KDM5B WT and mutants were detected by Western blotting using FLAG antibody. Center and right panels: Lysates were immunoprecipitated using FLAG antibody and the phosphoserine signal was detected by Western blotting. Normal rabbit IgG was used as a negative control. Input lanes represent 25% of the total protein. Figures are representative of at least 3 independent experiments.

In preparation for mass spectrometry analyses, recombinant cyclin B and CDK1 were incubated with purified GST-KDM5B in the presence of ATP. Reaction products were electrophoresed on a SDS-PAGE gel. The resulting gel was visualized with SYPRO Ruby, and gel bands were in-gel digested using trypsin prior to LC-MS analysis. Mass spectrometry analyses revealed S1328 as a putative phosphorylation site of CDK1 (Fig. 2a).

As mentioned above, in silico prediction of KDM5B residues phosphorylated by CDK1 was carried out using KinasePhos, and the highest scoring sites identified using KinasePhos were also selected for further study. Common properties of CDK1 recognition motifs include localization in loops or highly disordered regions^39^. Among the predicted phospho-acceptor sites, S1384 and S1456, are conserved across different vertebrate species and are located in disordered region (Fig. 2b). Putative phosphorylation sites identified via the two approaches, serines at 1328, 1384, and 1456 were substituted with alanines. While phosphorylation of KDM5B was detected in cells transfected with expression vectors for wild type and KDM5B^S1384A^, phosphorylation of KDM5B was attenuated upon mutation of S1328 or S1456 (Fig. 2c).

### Phosphorylation of KDM5B did not alter nuclear localization but attenuated target KDM5B occupancy and its ability to inhibit expression of pluripotency genes

**Figure 3:**
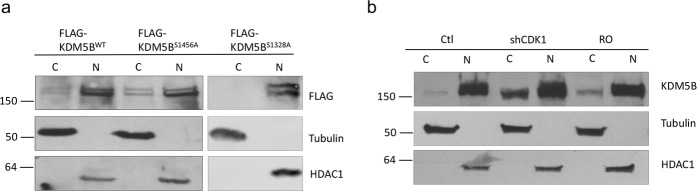
Phosphorylation of KDM5B by CDK1 does not significantly alter the nuclear localization of KDM5B. (**a**) Cell fractionation assay was conducted using lysates from MDA-MB-231 cells transfected with expression vectors for KDM5B^WT^, KDM5B^S1456A^, or KDM5B^S1328A^. The samples were analyzed by western blotting using the indicated antibodies. (**b**) Cell fractionation assay was conducted using lysates from MDA-MB-231 cells treated with RO3306 or infected with shCDK1 lentiviruses. The samples were analyzed by western blotting using the indicated antibodies. Tubulin served as cytoplasmic marker and HDAC1 served as nuclear marker. Figures are representative of at least 3 independent experiments.

It has been previously reported that AKT phosphorylated KDM5A, resulting in cytoplasmic retention of KDM5A. KDM5B was reported to be localized in cytoplasm during phases of the cell cycle phases wherein CDK1 is most active^19^. To investigate whether KDM5B phosphorylation by CDK1 alters KDM5B nuclear localization, subcellular fractionation was performed. Cytoplasmic localization of KDM5B^S1456A^ (which cannot be phosphorylated by CDK1) was slightly increased compared to the wild type (Fig. 3a). Increased cytoplasmic localization of endogenous KDM5B was observed in shCDK1-transfected cells (Fig. 3b). Pharmacological inhibition of CDK1 using RO3306 also resulted in increased cytoplasmic localization of KDM5B. However, in both cases the levels of nuclear KDM5B were not significantly altered. These data suggest that CDK1 plays a minor role in the regulation of nuclear localization of KDM5B.

**Figure 4:**
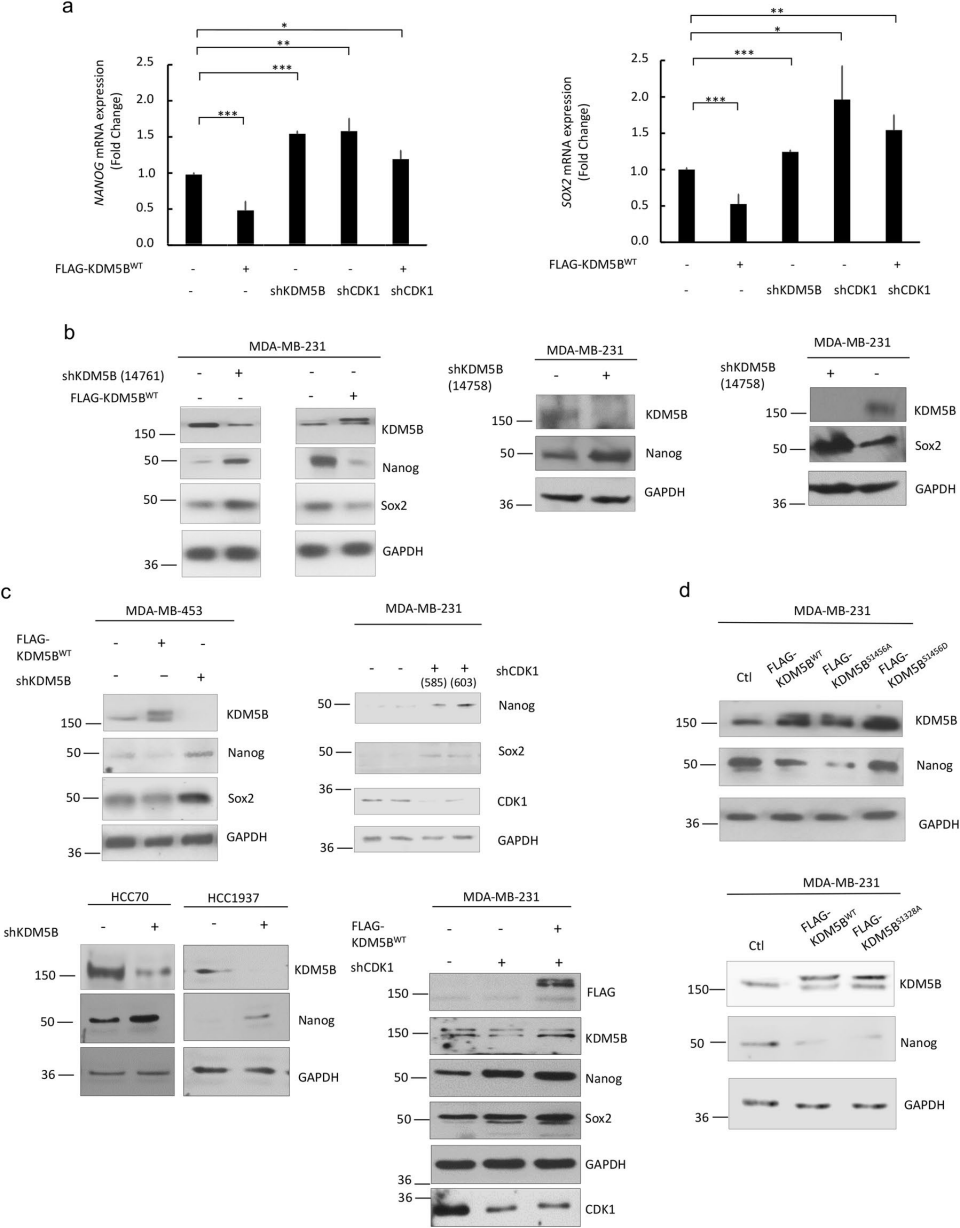
Phosphorylation of KDM5B perturbs the occupancy of KDM5B on *SOX2* and *NANOG* promoters. MDA-MB-231 cells were transfected as indicated with expression vectors for shCDK1, FLAG-KDM5B^WT^, FLAG-KDM5B^S1456A^, FLAG-KDM5B^S1456D^, or KDM5B^S1328A^. (**a**) ChIP analyses of lysates from MDA-MB-231 cells immunoprecipitated with antibodies against KDM5B, H3K4me3, or control non-specific rabbit immunoglobin, followed by PCR amplification of the promoter region of *SOX2* and *NANOG*. (**b**,**c**) ChIP analyses of lysates from MDA-MB-231 cells immunoprecipitated with antibodies against FLAG or control non-specific mouse immunoglobin, followed by PCR amplification of the promoter region of *SOX2* and/or *NANOG*. (**d**) Western blot analyses of cells transfected with expression vectors for FLAG-KDM5B^WT^, FLAG-KDM5B^S1456A^, FLAG-KDM5B^S1456D^, or KDM5B^S1328A^. Figures are representative of at least 3 independent experiments.

It has been reported that KDM5B binds to the promoters and regulate expression of the core pluripotency regulators, Sox2 and Nanog, in mouse embryonic stem cells^31^. High expression of Sox2 has been associated with the early stages of breast tumor initiation and the development of cancer stem cell properties^40^. To determine whether phosphorylation impacts KDM5B recruitment and histone modifications on *SOX2* and *NANOG* promoters, Chromatin IPs were performed. Downregulation of CDK1 resulted in enhanced recruitment of KDM5B and decreased H3K4me3 levels on *SOX2* and *NANOG* promoters (Fig. 4a). To determine whether the altered recruitment can be attributed to KDM5B phosphorylation, relative recruitment of FLAG-tagged KDM5B^WT^ and KDM5B^S1456A^ were determined using the FLAG antibody. Increased occupancy of KDM5B^S1456A^ was observed on both *SOX2* and *NANOG* promoters when compared to the KDM5B^WT^ cells (Fig. 4b). Mutation at S1328A did not alter KDM5B occupancy on the *SOX2* and *NANOG* promoters. Consistent with phosphorylation at S1456 resulting in perturbation of KDM5B recruitment to the promoter regions of pluripotency genes, the phosphomimetic mutant KDM5B^S1456D^ exhibited attenuated recruitment to the *NANOG* promoter (Fig. 4c). Altered recruitment of KDM5B^S1456A^ and KDM5B^S1456D^ when compared to KDM5B^WT^ cannot be attributed to differences in expression levels of mutant and WT KDM5B (Fig. 4d).

**Figure 5:**
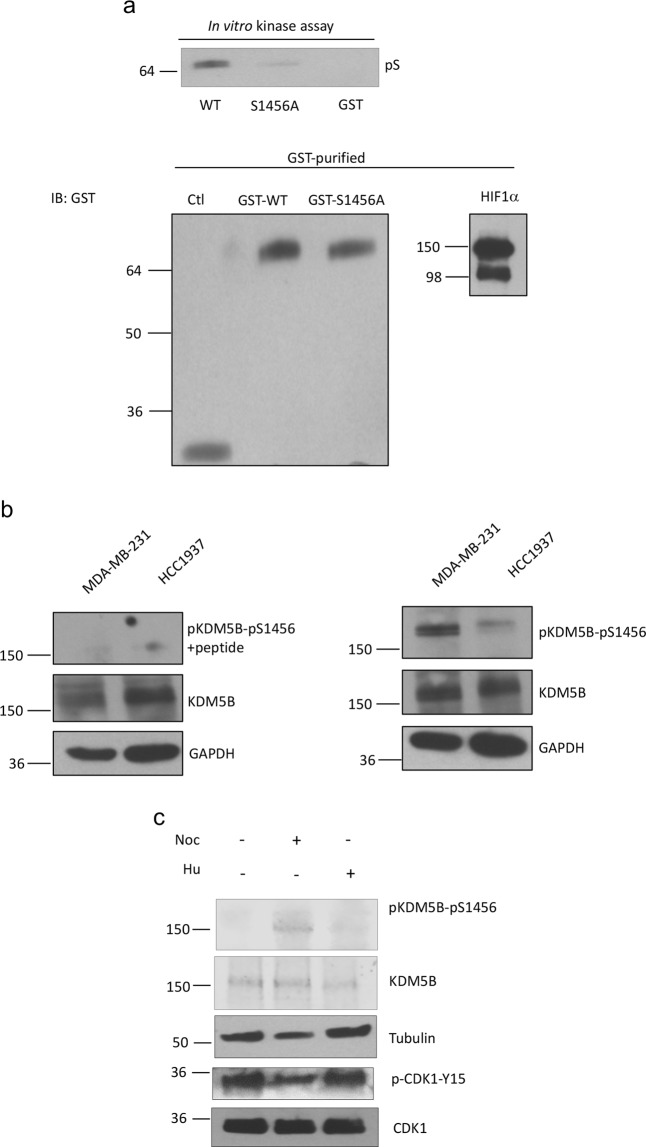
KDM5B mediates expression of pluripotency genes and role of CDK1 in KDM5B action. TNBC cells were transfected as indicated above with expression vectors for WT or mutant FLAG-KDM5B, shKDM5B, and/or shCDK1. Two clones each of shKDM5B (14758 and 14761) and shCDK1 (585 and 603) were used. (**a**) RNA was isolated and subjected to qRT-PCR to assess *SOX2* and *NANOG* mRNA levels using *GAPDH* as a control. (**b**–**d**) Western blotting analyses of endogenous and/or transfected KDM5B, CDK1, Nanog, and/or Sox2 relative to loading control (GAPDH) in TNBC cell lines. Figures are representative of at least 3 independent experiments.

To verify that CDK1 mediated phosphorylation of KDM5B regulates expression of pluripotency regulators, quantitative real time PCR and western blot analyses were conducted. Expression of both Sox2 and Nanog were significantly decreased in cells with increased KDM5B expression. Conversely, expression of Sox2 and Nanog were increased in shKDM5B-transfected MDA-MB-231 cells (Fig. 5a,b). Different clones of shKDM5B were used to verify the effect of KDM5B and to delimit the role off-target effects (Fig. 5b). Downregulation of KDM5B also increased Nanog expression in other TNBC lines, MDA-MB-453, HCC70 and HCC1937 (Fig. 5c). However, downregulation of KDM5B had the opposite effect on luminal epithelial breast cancer cell lines, MCF-7 and T47D, and resulted in downregulation of Nanog expression in these cells (Supplemental Fig. S1). We were unable to detect Sox2 in these cells. Our observation of divergent actions of KDM5B on Nanog expression in luminal epithelial cells and TNBC are consistent with a report by Yamamoto et. al. that supports differences in KDM5B function in mammary luminal and basal cells^21^. Their study also provides support for a role of KDM5B as a luminal lineage driving oncogene. It is possible that KDM5B contributes to the maintenance of the luminal cell lineage, while repressing the basal cell lineage.

Unexpectedly, when CDK1 expression was downregulated, Sox2 and Nanog levels were significantly increased when compared to control shRNA-transfected cells. Downregulation of CDK1 also attenuated KDM5B-induced decreases in Sox2 and Nanog expression (Fig. 5a,c). Our data support repressive roles for KDM5B and CDK1 in the expression of Sox2 and Nanog in MDA-MB-231 cells.

Consistent with enhanced ability of the phosphodefective mutant, KDM5B^S1456A^, to bind to the *NANOG* promoter, this mutant was the most effective at attenuating Nanog expression (Fig. 5d). Mutation at S1328 did not alter the ability of KDM5B to downregulate Sox2 and Nanog expression.

### CDK1-mediated phosphorylation of KDM5B is cell-cycle dependent

**Figure 6:**
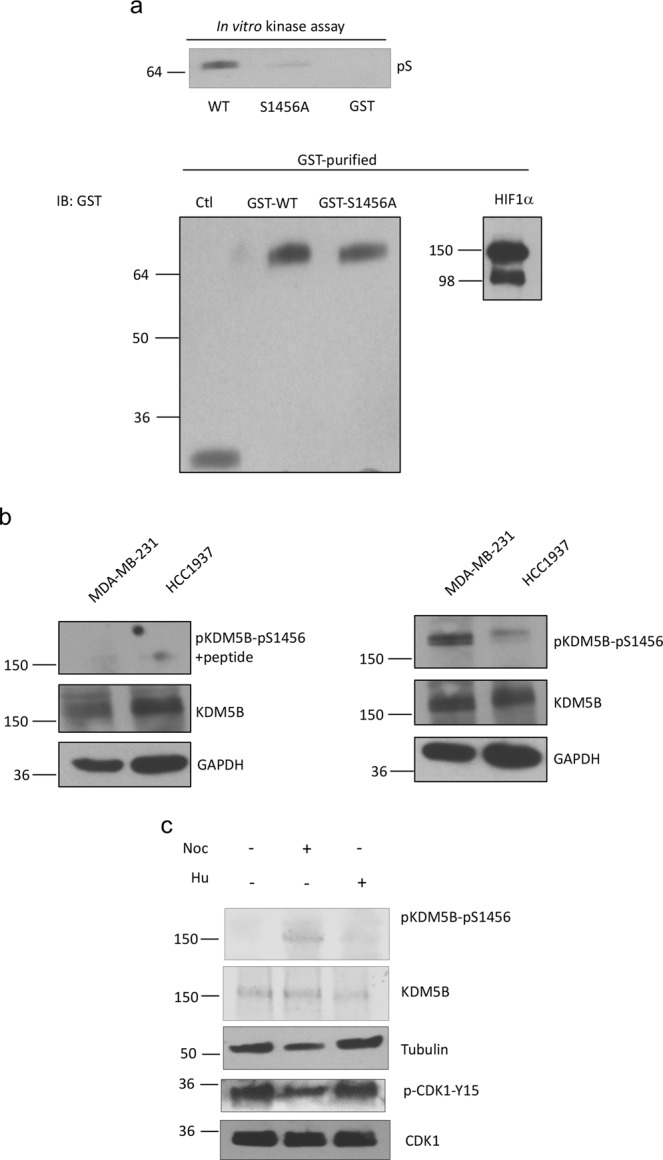
Phosphorylation of KDM5B is cell-cycle dependent. (**a**) Upper panel: *In vitro* kinase assay wherein recombinant CDK1 and cyclin B1 were incubated with purified GST-KDM5B^WT^ or GST-KDM5B^S1456A^. Phosphoserine signal was detected by Western blotting. Lower panel: Western blot analyses of purified GST-KDM5B^WT^ or GST-KDM5B^S1456A^ used in kinase assays. (**b**) Lysates from MDA-MB-231 and HCC1937 cells were analyzed by Western blotting using antibody against pKDM5B-S1456 in the absence or presence of phosphopeptide ERER[pS]YELVRSAETC. (**c**) Lysates from MDA-MB-231 cells treated with nocodazole (0.5 μg/ml, 18 hr) or hydroxyurea (2 mM, 12–16 hr) were analyzed by western blotting using indicated antibodies. Figures are representative of at least 3 independent experiments.

Because CDK1 is a key regulator of the cell cycle, we examined whether phosphorylation of KDM5B is altered during the phases of the cell cycle. For these studies our focus is on phosphorylation of S1456, because the phosphorylation of this residue was more relevant in the ability of KDM5B to regulate Nanog and Sox2 expression when compared to other residues. We first used *in vitro* and ELISA analyses to validate the phosphorylation of KDM5B at S1456 by CDK1, and to justify the generation of a S1456 phosphospecific antibody. The phosphorylation of KDM5B by CDK1 in an *in vitro* kinase was attenuated upon mutation at S1456 (Fig. 6a). These results were also evident from an ELISA assay (Supplemental Fig. S2) wherein GST antibody, which has been immobilized onto the solid surface of a 96-well plate, were complexed with *in vitro* kinase products, followed by the addition of the phosphoserine antibody. Phosphorylation of WT KDM5B, but not KDM5B^S1456A^, was evident from this colorimetric assay due to the ensuing interaction with HRP-conjugated secondary antibody, and the reaction between the HRP and the substrate tetramethylbenzidine, TMB.

Examination of cell cycle dependent phosphorylation of KDM5B was aided by the development of an antibody to detect KDM5B phosphorylated at S1456, pKDM5B-S1456. pKDM5B-S1456 was detected in MDA-MB-231 cells and another TNBC line HCC1937 (Fig. 6b). The signal detected by the phosphospecific antibody was attenuated upon incubation with phosphopeptide encompassing pKDM5B-S1456 and surrounding residues. Activation of CDK1 by treatment of cells with nocodazole (which arrests cells in G2/M phase) resulted in the increased levels of pKDM5B-S1456 (Fig. 6c). Treatment of cells with hydroxyurea, which arrested cells near the G1/S boundary, did not result in increased levels of pKDM5B-S1456. Together our studies provided further support for phosphorylation of KDM5B by CDK1.

### HEXIM1 attenuates KDM5B phosphorylation and is required for inhibition of the expression of pluripotency genes by KDM5B

**Figure 7:**
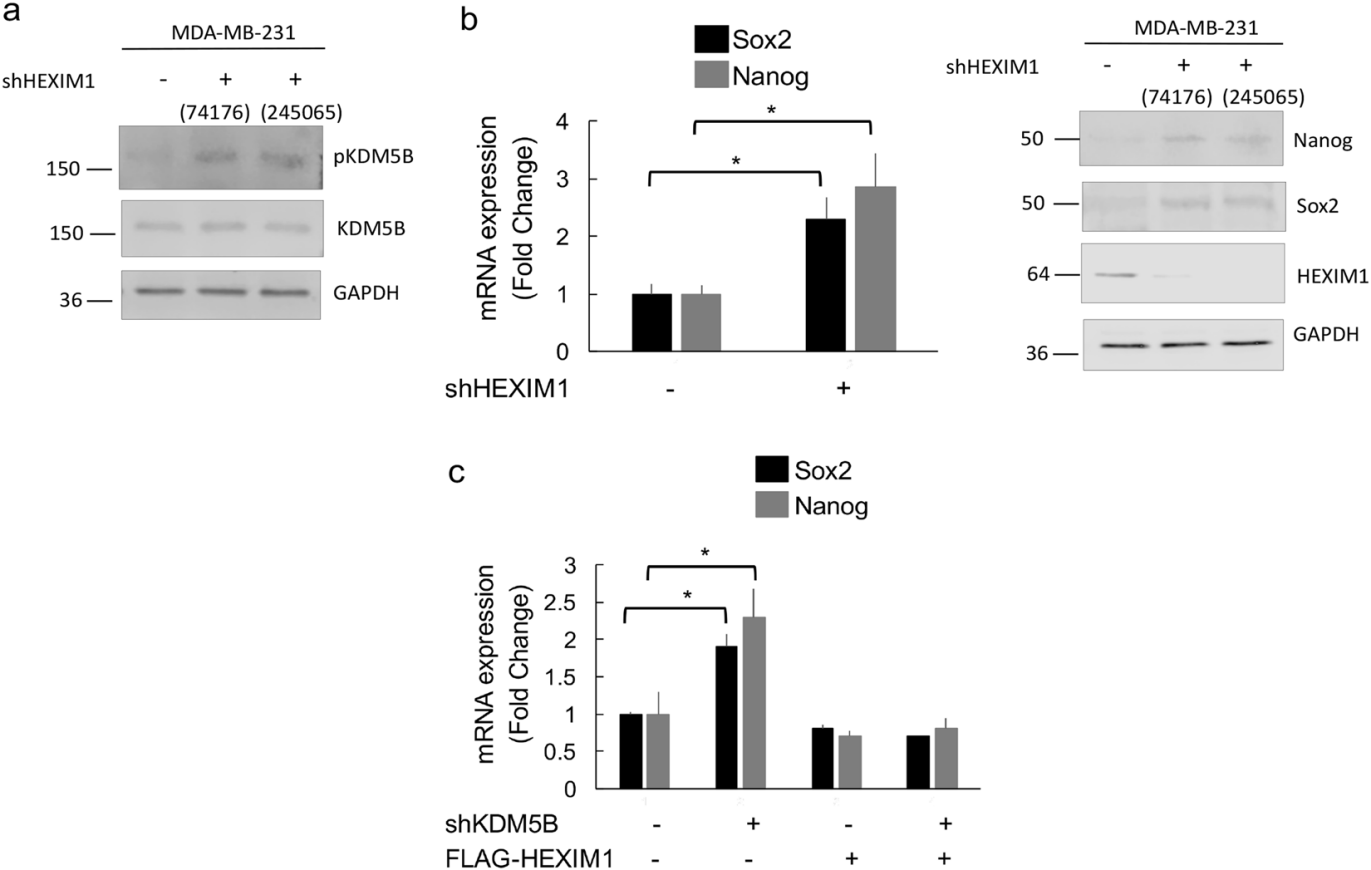
HEXIM1 counteracts is required for the inhibitory effects of KDM5B. MDA-MB-231 cells were transfected with expression vector for shHEXIM1, shKDM5B, and/or FLAG-HEXIM1. (**a**) Lysates were analyzed by Western blotting using indicated antibodies. (**b**) RNA was isolated and subjected to qRT-PCR or lysates were analyzed by Western blotting for expression of indicated genes or proteins, respectively. (**c**) RNA was isolated and subjected to qRT-PCR analyses of *SOX2* and *NANOG* mRNA levels using *GAPDH* as a control. Figures are representative of at least 3 independent experiments.

We previously reported that the tumor suppressor HEXIM1 is a mediator of KDM5B recruitment to its target genes, and HEXIM1 is required for the inhibition of nuclear hormone receptor activity by KDM5B^25^. We also reported that induction of HEXIM1 expression by a small molecule inducer resulted in downregulation of Nanog expression and CSC fraction in MDA-MB-231 cells^41^. We determined if HEXIM1 is also a mediator of the regulation of the expression of pluripotency genes by KDM5B. Downregulation of HEXIM1 resulted in increased levels of pKDM5B-S1456 (Fig. 7a). Consistent with KDM5B phosphorylation as attenuating KDM5B occupancy and ability to inhibit expression of pluripotency genes, downregulation of HEXIM1 resulted in increased expression of Sox2 and Nanog (Fig. 7b). Moreover, ectopic expression of HEXIM1 rescued the effects of KDM5B downregulation, resulting in downregulation in expression of *Sox2* and *Nanog* (Fig. 7c). These data further support the role of HEXIM1 as a critical mediator of the effects of KDM5B.

### KDM5B reduces the cancer stem cell frequency in TNBC

**Figure 8:**
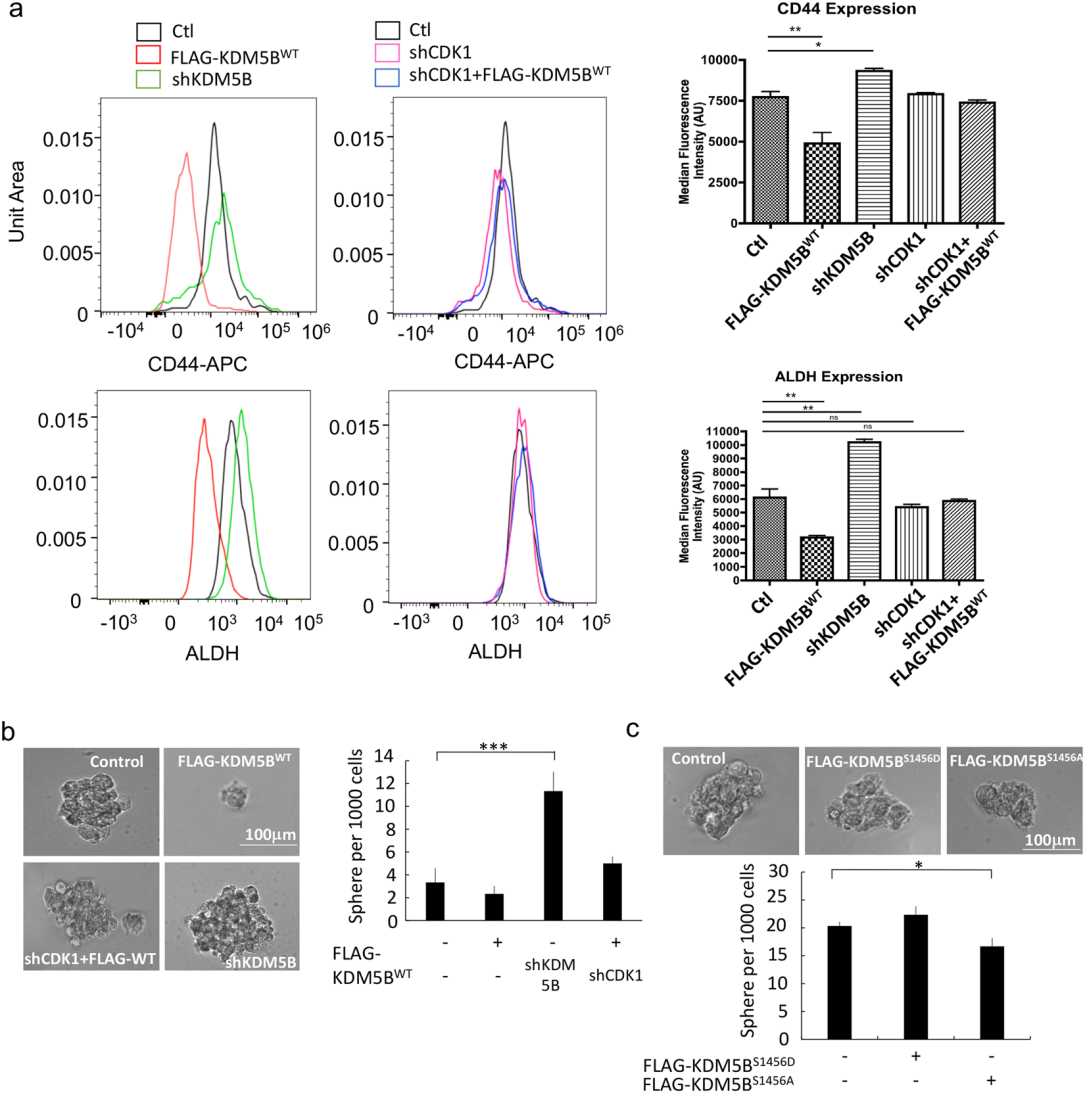
KDM5B induces a reduction in the CSC population in TNBC and role of CDK1 in KDM5B action. MDA-MB-231 cells were transfected as indicated with expression vectors for FLAG-KDM5B^WT^, shKDM5B, and/or shCDK1. Shown are (**a**) flow cytometry analyses of ALDH^+^ (aldehyde dehydrogenase) and CD44-APC (allophycocyanin) positive populations including mean fluorescence values (MFI) and (**b**) mammosphere formation assay. (**c**) MDA-MB-231 cells were transfected with expression vectors for FLAG-KDM5B^WT^, -KDM5B^S1456A^ or -KDM5B^S1456D^. Transfected cells were cultured in mammosphere media and colonies were quantified. Figures are representative of at least 3 independent experiments.

The regulation of the expression of the pluripotency regulators by CDK1 and KDM5B prompted us to investigate whether the cancer stem cell population is regulated by CDK1 and KDM5B. The CD44^+^/ALDH^+^ population was reduced in cells with increased KDM5B expression, and this reduction was reversed upon downregulation of CDK1 expression (Fig. 8a). Although the CD24^-^ population was not altered by KDM5B (data not shown), mammosphere formation was regulated by KDM5B, and this action was dependent on the presence of CDK1 (Fig. 8b). Mammosphere formation was regulated by KDM5B in other TNBC lines, MDA-MB-453 and HCC1937 (Supplemental Fig. S3). Phosphodefective mutant KDM5B^S1456A^ was more effective at inhibiting mammosphere formation than the phosphomimetic mutant KDM5B^S1456D^ (Fig. 8c).

We also assessed the functional role of KDM5B on TNBC cell invasion. We observed reduced invasion of MDA-MB-231 cells with increased KDM5B expression (Supplemental Fig. S4). Conversely, downregulation of KDM5B resulted in increased cell invasion. This effect was attenuated upon downregulation of CDK1 expression.

### CDK1 also induces recruitment of KDM4B, which has inhibitory effects on Nanog expression and mammosphere formation

We then determined the basis for the unexpected finding that CDK1 inhibited expression of pluripotency genes and cancer stem cell frequency, despite inhibiting KDM5B recruitment to *NANOG*. A possibility is that KDM5B recruitment and attenuation of H3K4me3 levels on *SOX2* and *NANOG* may not be the critical determinants of KDM5B regulation of the expression of pluripotency genes. However, this is unlikely because of the enhanced recruitment and repressive effects of KDM5B^S1456A^, indicating that CDK1-induced phosphorylation of KDM5B attenuated the inhibitory effects of KDM5B on the CSC population.

We examined the possibility that CDK1 induced recruitment of a transcription factor that inhibits Sox2 and Nanog expression, counterbalancing the negative effects of CDK1 on KDM5B recruitment. Another histone demethylase, KDM4B, has been shown to inhibit transcription by attenuating H3K36 methylation. CDK1 interacted with KDM4B and influenced recruitment of KDM4B to *Nanog* (Supplemental Fig. S5). Downregulation of CDK1 resulted in decreased KDM4B occupancy of *Nanog*, which can be correlated with increased H3K36me3 levels. An inhibitory effect of KDM4B on expression of Nanog is supported by our data that downregulation of KDM4B resulted in increased H3K36me3 levels on *Nanog* and increased Nanog expression. We did not find evidence for regulation of Sox2 expression by KDM4B. It is possible that other transcription factors, in addition to KDM5B, with similar functions as KDM4B are involved in the regulation of Sox2 expression by CDK1.

## Discussion

Our findings support the repressive effects of KDM5B on the expression of pluripotency genes and the CSC population. CDK1 influenced this function by phosphorylating KDM5B, resulting in attenuated recruitment of KDM5B to pluripotency genes.

Patients with TNBC have poor clinical outcomes and limited therapeutic options^3,42^. The CD44^+^/CD24^−^/ALDH^+^ population is enriched in certain TNBC subtypes^6,10,11^, and CSCs are proposed to contribute to worse progression-free survival in TNBC patients^6,43^. CSCs are considered as major contributors to therapeutic resistance and metastasis of malignant breast cancer cells^44,45^. Chemotherapies have been demonstrated to stimulate CSC proliferation^46^, supported by the inhibition of mitotic kinase leading to the recurrence and enrichment of the CSC^47,48^. The high degree of plasticity in CSCs is maintained partly by reversible epigenetic modifications, including DNA methylation^49,50^.

Epigenetic modifications are known to play critical roles in the activation and repression of genes important for cell differentiation^51,52^. Perturbation of the balance among epigenetic modifications results in dysregulation of cancer related genes^53^. Published reports support a role for KDM5B in the maintenance or differentiation of normal and cancer stem cells^31,54,55^. In oral cancers, KDM5B enables the transition of cells into a stem-cell like phenotype^56^. Melanoma cells expressing high levels of KDM5B cycled slowly and possessed a high self‐renewal potential, while knockdown of KDM5B resulted in a reduction in tumorigenic activity^57^. On the other hand, KDM5B suppressed leukemogenesis and induced Acute Myeloid Leukemia (AML) cells to differentiate out of the leukemia stem cell compartment^58^. Other support for the pro-differentiation role of KDM5B include reports that knockdown of KDM5B in mESCs results in failure of neural differentiation and inability to efficiently silence stem- and germ-cell-related genes^59^. Depletion of KDM5B also impairs ESC differentiation to embryoid bodies, causing partial retention in a self-renewing stage. While conflicting, these findings support a role for KDM5B in cell fate decision. Our findings are consistent with a role for KDM5B in the reduction of the stem cell population in TNBC.

KDM5B is overexpressed in certain cancers, and has led to interest in the development of KDM5B inhibitors for clinical use^60,61^. In breast cancer, increased KDM5B expression drives a luminal cell-specific expression program^21,62^. Our data are consistent with the inhibition of basal-like cell enrichment by KDM5B^24^, given that a majority of TNBC belong to the basal subtype^2^. Along this line, KDM5B inhibited Sox2 expression that has been proposed to play a role in the development of a less differentiated phenotype of the basal subtype, with Sox2 expression being observed in 43% of basal-like breast carcinomas^63^.

While an oncogenic role for KDM5B in the luminal subtype of breast cancer has been reported, there are also reports of tumor suppressor action of KDM5B. Overexpression of KDM5B inhibited MDA-MB-231 cell migration and invasion by interacting with LSD1/NuRD complexes^23^. On the other hand, KDM5B has been reported to promote MDA-MB-231 cell migration and invasion by regulating MALAT1 and has-miR-448 expression^64^. However, the KDM5B species reported in this study does not appear to be full-length, and may complicate the interpretation of their findings on the effect of KDM5B on cell invasion. The conflicting reports on KDM5B action in cancer cells may be attributed to KDM5B target genes and interacting partners, which play diverse roles in development, differentiation, and the cell cycle^15,30^. Our data provide further support for HEXIM1 as critical in determining KDM5B function.

Post-translational modifications of histone methylases and demethylases have been receiving more attention. SKP2 inactivates KDM5B through ubiquitination in prostate cancer cells^65^. SUMOylation of KDM5B results in altered KDM5B occupancy at certain genes in HEK293T cells^66^. In luminal breast cancer cells, activated PI3K/Akt pathway phosphorylates another member of the KDM5B family, KDM5A, which results in increased localization of KDM5A to the cytoplasm and enhancement of its oncogenic role^67^. On the other hand, nuclear KDM5A functions as a tumor suppressor by exerting its demethylase activity^68^. Based on our observations, phosphorylation of KDM5B attenuates it ability to regulate expression of pluripotency genes and downregulate the CSC population. Whether the phosphorylation status of KDM5B results in different interaction partners and/or results in a conformational change in KDM5B that attenuate recruitment to target genes are subjects of our current investigation.

We observed that CDK1 phosphorylated KDM5B, which can be correlated with decreased recruitment to the promoter of *SOX2* and *NANOG*. Thus, increased Sox2 and Nanog expression and the attenuation of the repressive effects of KDM5B on the expression of these pluripotency genes upon downregulation of CDK1 appears paradoxical. The paradoxical finding on CDK1 function may also be attributed to other targets of CDK1^69^, which may counteract repressive actions of CDK1 on KDM5B. Our data suggest that this paradox may be attributed partly to CDK1-induced recruitment of another histone modifier, KDM4B, that has inhibitory effects on Nanog expression. Thus, the relative levels of KDM5B and KDM4B may determine how CDK1 influences the stem cell fraction.

While CDK1 has a well-known role in promoting cell proliferation, reports indicate divergent actions. CDK1 has been shown to inhibit the phosphorylation of PDK1, subsequently activating the PI3K/Akt pathway, and resulting in the development of pluripotency^70^. On the other hand, CDK1 phosphorylation inhibited the oncogenic activity of TAZ, a transcription factor that confers stem cell-like properties in breast cells^71^. CDK1 promoted mesenchymal stem cell differentiation in osteoblasts through phosphorylation of EZH2 and inhibition of its methyltransferase activity^72^. Divergent actions of CDK1 may contribute to the modest activity of CDK1 inhibitors exhibited in clinical trials^73^. This modest activity may be attributed to the development of drug resistance, which can be attributed to the CSC population. Our findings suggest a contributing factor to the unsatisfactory outcomes in clinical trials of CDK1 inhibitors.

## Conclusions

In this study, we discovered that protein kinase CDK1 phosphorylates KDM5B at S1456, resulting in decrease occupancy of KDM5B on the promoter regions of pluripotency genes, leading to the suppression of H3K4 trimethylation. KDM5B inhibits the expression of these genes, which can be correlated with a reduction in the CD44^+^/CD24^−^/ALDH^+^ population in TNBC. Preclinical evidence suggests that CD44^+^/CD24^−^/ALDH^+^ population in TNBC is correlated with worse progression-free survival. Our findings provide insight into the pros and cons of targeting KDM5B or CDK1, in particular with regards to the development of drug resistance.

## Methods

### Cell culture, transfections and lentiviral infections

TNBC lines were obtained from the American Tissue Culture Collection on April 2017, and were maintained based on the instructions from ATCC. Cells were transfected with control or expression vector for FLAG-KDM5B (WT or mutants) using FuGENE HD (Promega) according to the manufacturer’s instructions. For treatment with Vx-11e and RO3306, concentrations of 100 μM and 10 μM were used, respectively. Plasmids used to generate KDM5B shRNA (shKDM5B), CDK1 shRNA (shCDK1) and KDM4B shRNA (shKDM4B) lentiviruses were obtained from Sigma-Aldrich. To generate lentiviral particles, 293FT cells were transfected with envelope expressing plasmid (pMD2.G), packaging plasmid (psPAX2) and shRNA expression plasmid (pLKO) using Lipofectamine 2000. Media containing lentiviral particles were collected 48–72 h post-transfection and used to transduce breast cancer cells for 12–16 hours. Puromycin was then used to select for cells expressing shRNAs.

### Co-immunoprecipitations

Endogenous proteins were co-immunoprecipitated and analyzed as previously described^25^ and described in more detail in the Supplementary Methods.

### Site-directed mutagenesis

pCMX-FLAG plasmid was derived from the pCMV mammalian expression plasmid^74^. Wild-type KDM5B cDNA was cloned into the pCMX plasmid. KDM5B mutants were generated using the QuikChange II XL Site-Directed Mutagenesis Kit according to the manufacturer’s protocol (Clontech; Palo Alto, CA). Primers sequences are provided in Table S1.

### GST constructs and purification

To generate constructs for bacterial expression of wild-type and S1456A KDM5B in frame with glutathione-S-transferase (GST), cDNAs encoding C-terminal of KDM5B (1156–1544) were subcloned into the pGEX-4T vector. GST-KDM5B (wild-type and mutant) proteins were inducibly expressed in *Escherichia coli* strain BL21 and purified using Glutathione Sepharose beads (Amersham Biosciences). Expression of GST proteins were detected using GST monoclonal antibody (catalog number 2624, Cell Signaling Technology).

### *In vitro* kinase assay

Recombinant cyclin B and CDK1 (0.1 μg, catalog number 14-450-D, Millipore) were incubated with 1 μg of purified GST-KDM5B (wild-type or mutant) in the presence of 100 μM ATP in kinase buffer (50 mM β-glycerophosphate, pH 7.4; 10 mM MgCl2; 10 mM NaF; 1 mM DTT) for 30 min at 30 °C. Reactions were stopped by adding equal volume of 10 mM EDTA and were kept in −20 °C until further analysis. Reaction products were analyzed using ELISA (as described below) or Western blotting and detected using phosphoserine antibody (catalog number AB1603, Millipore).

### Determination of KDM5B phosphorylation sites via mass spectrometry

The *in vitro* phosphorylated KDM5B was separated on a SDS-PAGE gel and the protein band was visualized by Sypro Ruby. A band from the SDS-PAGE gel corresponding to KDM5B was excised, the protein was in-gel digested using trypsin^75^, and the digests were analyzed by data-dependent LC-MS/MS with collision-induced dissociation (CID) using a Thermo Fisher Scientific Fusion Lumos mass spectrometry system^76^. The HPLC column was a Dionex 15 cm × 75 μm id Acclaim Pepmap C18, 2 μm, 100 Å reversed-phase capillary chromatography column. The peptides were eluted from the column by an acetonitrile/0.1% formic acid gradient at a flow rate of 0.3 μL/min. The acquired mass spectrometry data was analyzed using MassMatrix database search software (Version 3.10, MassMatrix, Columbus, OH) to identify phosphorylated peptides and all the possibly phosphorylated peptides were manually verified by inspecting their MS/MS spectra.

### ELISA assay

Ninety-six-well plates were coated (overnight, 4 °C) with 100 ng/well of GST monoclonal antibody (catalog number 2624, Cell Signaling Technology) and incubated with blocking buffer (2% BSA, 0.1% Tween 20) for 1 h. Serially diluted kinase products (generated as described above) were loaded onto the coated wells and incubated for 2 hours at room temperature, followed by the addition of 100 μl of phosphoserine antibody (1:500, catalog number 06-427, Millipore) and incubation for an additional 2 hours at room temperature. HRP-conjugated goat anti-rabbit IgG (1:10,000) in blocking buffer was added and incubated for 1 h at room temperature. Reaction products were detected by the addition of 100 μl chromogenic substrate (3,3’,5,5’- tetramethylbenzidine, TMB) for 30 min.

Plates were washed five times with washing buffer (PBS, pH 7.4, containing 0.02% (v/v) Tween 20). The reaction was stopped with 50 μl of 1 M HCl and absorbance at 450 nm was measured with reduction at 630 nm using an ELISA plate reader.

### Cell fractionation assay

Cell were harvested and washed with 1X PBS, pelleted, and resuspended in cytosolic extraction buffer (CEB, 10 mM HEPES, 3 mM MgCl_2_, 20 mM KCl, 5% Glycerol, 0.5 mM DTT, 0.5% NP40, protease inhibitors and phosphatase inhibitors). Cytosolic extracts were obtained by passing the suspension through a 25-gauge needle ten times and by centrifugation at 6000 rpm for 10 min at 4 °C. The pellets were washed with CEB three times and resuspended in nuclear extraction buffer (NEB, 20 mM HEPES, 3 mM MgCl_2_, 225 mM NaCl, 1 mM EDTA, 10% Glycerol, 0.5 mM DTT, 0.5% NP40, protease inhibitors and phosphatase inhibitors). Nuclear extracts were obtained by passing the suspension through a 26-gauge syringe needle ten times and by centrifugation at 14,000 rpm for 10 min at 4 °C.

### Chromatin immunoprecipitation and ChIP qRT-PCR

Cells were grown in 150 mm diameter dishes and processed for ChIP analyses as described previously^77^, and described in more detail in the Supplementary Methods. Primers sequences are provided in Table S1^78,79^.

### Real-time quantitative RT-PCR

Total mRNAs were extracted using TRIzol® reagent (Invitrogen) according to the manufacturer’s protocol. mRNAs were reverse transcribed using the M-MLV Reverse Transcriptase kit (Invitrogen) following the recommended protocol. Quantitative RT-PCR (qRT-PCR) reactions were performed in duplicate with SYBR Green Master Mix (catalog number 170-8882, Bio-Rad) and 10 μM forward and reverse primers. Products were then subjected to melting curve analysis. Gene expression was quantified relative to the expression of the housekeeping gene, glyceraldehyde-3-phosphate dehydrogenase (*GAPDH*). Primer sequences are provided in Supplementary Table 1, some of which are based on previous reports^80–82^.

### Western blotting

Cell lysates were analyzed by Western blotting as described previously^83^, and described in more detail in the Supplementary Methods.

### Generation of phosphorylated KDM5B, pKDM5B-S1456, antibody

Phosphopeptide ERER[pS]YELVRSAETC was coupled with keyhole limpet hemocyanin by m-maleimidobenzoic acid N-hydroxysuccinimide ester method. The conjugated phosphopeptide was used to immunize rabbits. The antibody is double purified from antiserum to ensure specificity. The crude antiserum is passed through a first column coupled with the phosphopeptide. The sequence specific and phosphate sequence specific antibodies recovered from the first purification were applied onto a second column coupled with non-phosphorylated peptide. Phosphospecific antibodies recovered in the flow through were used in ELISA tests with 2 peptides, the free phosphopeptide and non-phosphopeptide (to test for cross reaction). The antibody was validated by analyzing lysates from TNBC cells using Western blotting with pKDM5B-S1456 antibody in the absence or presence of 10 μM of phosphopeptide.

### Flow cytometry and aldehyde dehydrogenase assay

Cells were dissociated using Accutase (Innovative Cell Technologies, catalog number AT106-550) and filtered through 40 micron filters. Cells (500,000) were then washed with FACS buffer (2% FBS, 0.2% sodium azide solution in 1x PBS) and incubated with anti-CD44 conjugated to allophycocyanin (APC, catalog number 560–890, BD Pharmingen^TM^) and anti-CD24 conjugated to Fluorescein isothiocyanate (FITC, catalog number 560–992, BD Pharmingen^TM^) for 30 minutes on ice. Isotype-matched conjugated non-immune antibodies were used as negative controls (APC-ISO, catalog number 17-4321-81; FITC-ISO, catalog number 11-4321-81, Thermo Fisher Scientific). Cells were then washed with FACS buffer, resuspended in fixative solution (3.7% PFA in 1X PBS), and incubated overnight at 4 °C before analysis by flow cytometry (BD LSR Fortessa). Data analysis was completed using FlowJo Software v10.

Aldehyde Dehydrogenase assays were performed using the ALDEFLUOR Assay Kit (StemCell Technologies, catalog number 01700) as described in the manufacturer’s protocol. Briefly, 10^6^ cells were centrifuged and resuspended in 1 mL ALDH assay buffer, followed by addition of substrate BODIPY-aminoacetaldehyde. A portion of each reaction was transferred into a tube containing ALDH inhibitor, Diethylaminobenzaldehyde, to control for background fluorescence. Cells were then incubated for 40 min at 37 °C. Levels of ALDH expression were analyzed by flow cytometry and FlowJo Software v10.

### Mammosphere assay

Cells were sorted using BD FACS Aria II and cultured in duplicate rows of serial dilutions into a 96-well plate and incubated for 10–14 days in DMEM media supplemented with 2% B27 (Invitrogen) in the presence of 20 ng/mL EGF and 10 ng/mL bFGF. Mammosphere-forming efficiency was calculated as the number of spheres divided by the number of single cells seeded.

### Cell invasion assay

Transwell migration assay were performed as previously described^25^ and described in more detail in the Supplementary Methods.

### Data analyses

Statistical significance was determined using Student’s *t* test comparison for unpaired data.

## Supplementary information

Supplementary info

## Data Availability

The authors declare that all the materials, data and associated protocols related to this manuscript are available upon reasonable request.
