# Supplementary material for: Phosphorylation of the histone demethylase KDM5B and regulation of the phenotype of triple negative breast cancer
Source: Sci Rep. 2019 Nov 27;9:17663. doi: 10.1038/s41598-019-54184-0 (PMC6881367; doi:10.1038/s41598-019-54184-0)

Phosphorylation of the histone demethylase KDM5B and regulation of the phenotype of triple negative breast cancer

I-Ju Yeh, Emily Esakov, Justin D. Lathia, Masaru Miyagi, Ofer Reizes, Monica M. Montano

SUPPLEMENTARY METHODS

Co-immunoprecipitations— MDA-MB-231 cells were lysed using RIPA buffer (20 mM Tris/HCl, pH 7.5, 150 mM NaCl, 0.5% sodium deoxycholate, 1% Igepal, 1 mM EDTA and 0.1% SDS) and were incubated with Protein G beads that had been pre-adsorbed with antibodies against phosphoserine (catalog number AB1603, Millipore), KDM5B (catalog number A301-813A, Bethyl Laboratories), KDM4B (catalog number A301-477A, Bethyl Laboratories), FLAG (catalog number F1804, Sigma-Aldrich), CDK1, or non-specific Rabbit/Mouse IgG (as negative controls). The beads were washed with RIPA buffer and PBS twice. After the final wash, immunoprecipitated proteins were eluted using SDS sample buffer, and analyzed by SDS-PAGE electrophoresis. Western blot analyses were performed as described below.

Chromatin Immunoprecipitation and ChIP qRT-PCR— Cells grown in 150 mm diameter dishes were fixed with 1% formaldehyde and lysed in SDS-lysis buffer with protease inhibitors. Lysed cells were sonicated using a Branson 450 sonicator. Clarified sonicated chromatin was diluted 10-fold in ChIP dilution buffer and used for immunoprecipitation with antibody. The antibody–chromatin complexes were bound with Protein G beads and subjected to a series of washes. The antigen–DNA complexes were eluted, reverse cross-linked overnight at 65°C, and the DNA purified using phenol/chloroform extraction. Ethanol precipitated pellets were resuspended in water and used as a template for PCR analysis. qRT-PCR reactions were performed in duplicate with SYBR Green Master Mix (catalog number 170-8882, Bio-Rad) and 10 μM of forward and reverse primers. Products were subjected to melting curve analysis. The fold enrichment values for known KDM5B binding sites were determined by normalizing to enrichment values for the control site.

Western Blotting—Total protein was extracted using MPER Mammalian Protein Extraction reagent (Thermo Fisher Scientific). Proteins were detected using their respective primary antibodies and HRP (horseradish peroxidase)-conjugated secondary antibody. GAPDH or tubulin were used as loading controls. Signals were visualized using the LI-COR Odyssey System, and quantified using the Image J software. Signals in each case were normalized to their respective GAPDH or tubulin values to calculate the relative expression levels.

Cell Invasion Assay. Transwell inserts (8-mm-diameter pore size; Corning Costar) were coated with 10% MatrigelTM and placed inside the wells of a 24-well plate. MDA-MB-231 cells transfected with control vector, FLAG-KDM5B, shKDM5B, and/or shCDK1 suspended in Opti-MEM® (serum-free culture medium, Invitrogen) were aliquoted onto the upper chamber of the Transwell insert (50,000 cells/well). The lower chambers contained MEM (minimal essential medium) supplemented with 5% FBS. The cells were allowed to migrate to the lower chamber at 37°C for 48 h. After incubation, cells invading the MatrigelTM were fixed with 3% paraformaldehyde and stained with 0.5% crystal violet. Invading cells were counted from five random fields per well under a microscope.

SUPPLEMENTARY FIGURE LEGENDS

Figure S1. Regulation of pluripotency genes by KDM5B in non-TNBC lines. MCF-7 and T47D cells were transduced with shKDM5B lentiviruses. Shown are western blotting analyses of endogenous and/or transfected KDM5B and Nanog relative to loading control (GAPDH). Figures are representative of at least 3 independent experiments.

Figure S2. Detection of the phosphorylation of KDM5B by an ELISA assay. Serially diluted products from an *in vitro* kinase assay were loaded onto GST coated wells and incubated with phosphoserine antibody and HRP-conjugated goat anti-rabbit. Signals were detected by the addition of chromogenic substrate (TMB) followed by quantification of absorbance at 450 nm wavelength. GST-WT is $KDM5B^{WT}$ conjugated with GST, while GST-S1456A is $KDM5B^{S1456A}$ conjugated with GST. GST-Control (GST-Ctl) served as negative control, while GST-HIF1α as positive control. Figures are representative of at least 3 independent experiments.

Figure S3. Mammosphere formation assay in MDA-MB-453 and HCC1937 cells. MDA-MB-453 and HCC1937 cells were transfected as indicated with expression vectors for FLAG-$KDM5B^{WT}$ or shKDM5B respectively. Transfected cells were cultured in mammosphere media and colonies were quantified. Figures are representative of at least 3 independent experiments.

Figure S4. Regulation of cell invasion by KDM5B and CDK1. MDA-MB-231 cells were transfected with control vector (Ctl), or expression vectors for $KDM5B^{WT}$, shKDM5B, and/or shCDK1. Invasion of cells through MatrigelTM was assessed in the Transwell invasion assay. Figures are representative of at least 3 independent experiments.

Figure S5. Involvement of another histone demethylase, KDM4B, in the regulation of Nanog by CDK1. (A) Lysates from MDA-MB-231 cells were immunoprecipitated using antibodies against KDM4B or CDK1 and analyzed for co-immunoprecipitation of CDK1 or KDM4B by Western blotting. Normal rabbit IgG was used as a negative control. Input lanes represent 25% of the total protein. MDA-MB-231 cells were transfected with control vector or expression vector for shCDK1. (B) ChIP analyses of lysates from MDA-MB-231 cells immunoprecipitated with antibodies against KDM4B, H3K36me3, or control non-specific rabbit immunoglobin, followed by PCR amplification of the promoter region of *SOX2* and *NANOG*. Figures are representative of at least 3 independent experiments. (C) Western blotting analyses of endogenous KDM4B, Nanog, and Sox2 relative to loading control (GAPDH). (D) MDA-MB-231 cells were transfected with expression vector for shKDM4B. Transfected cells were cultured in mammosphere media and colonies were quantified. Figures are representative of at least 3 independent experiments

Supplementary Table S1. Primer sequences

S1

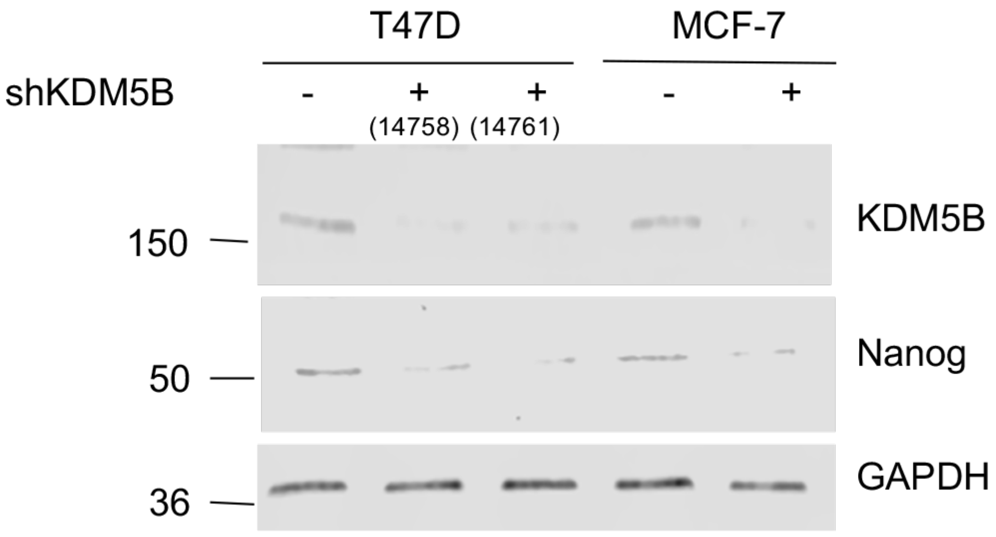

S2

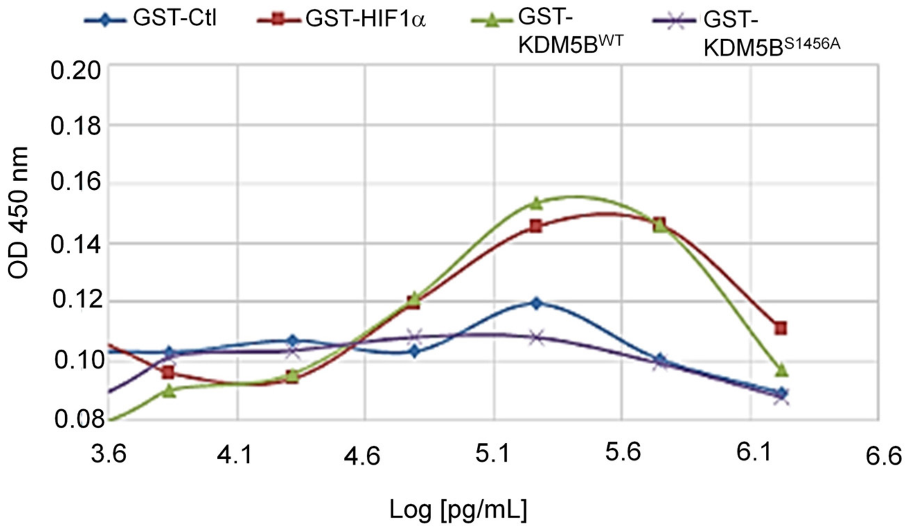
GST-Ctl
GST-HIF1α
GST-KDM5B^WT
GST-KDM5B^S1456A
0.20
0.18
0.16
0.14
0.12
0.10
0.08
OD 450 nm
3.6
4.1
4.6
5.1
5.6
6.1
6.6
Log [pg/mL]

S3

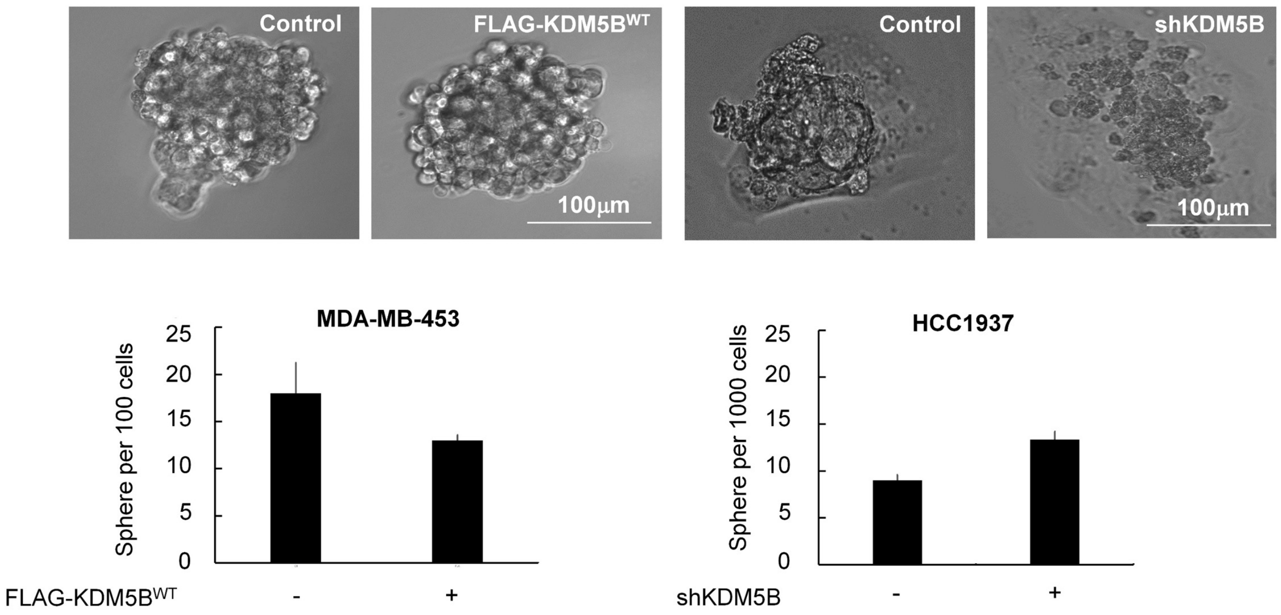
Control
FLAG-KDM5B^{WT}
100μm
Control
shKDM5B
100μm
MDA-MB-453
Sphere per 100 cells
25
20
15
10
5
0
FLAG-KDM5B^{WT}
-
+
HCC1937
Sphere per 1000 cells
25
20
15
10
5
0
shKDM5B
-
+

S4

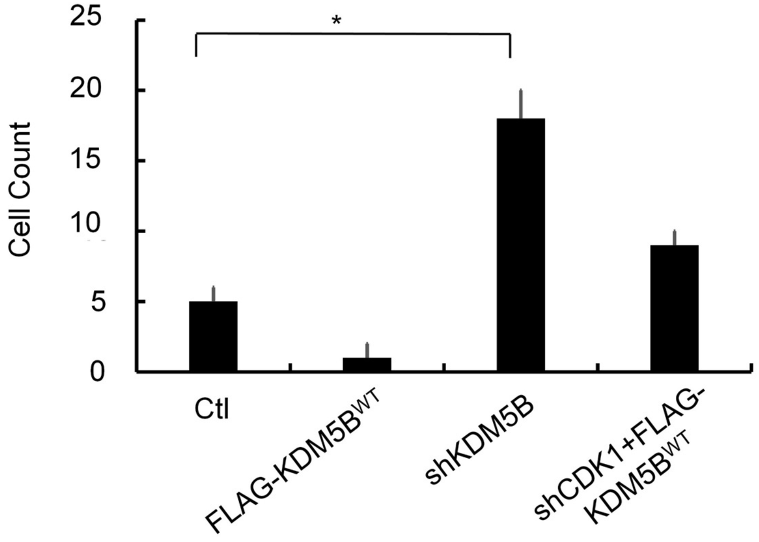
25
20
15
10
5
0
Cell Count
*
Ctl
FLAG-KDM5B^{WT}
shKDM5B
shCDK1+FLAG-KDM5B^{WT}

S5

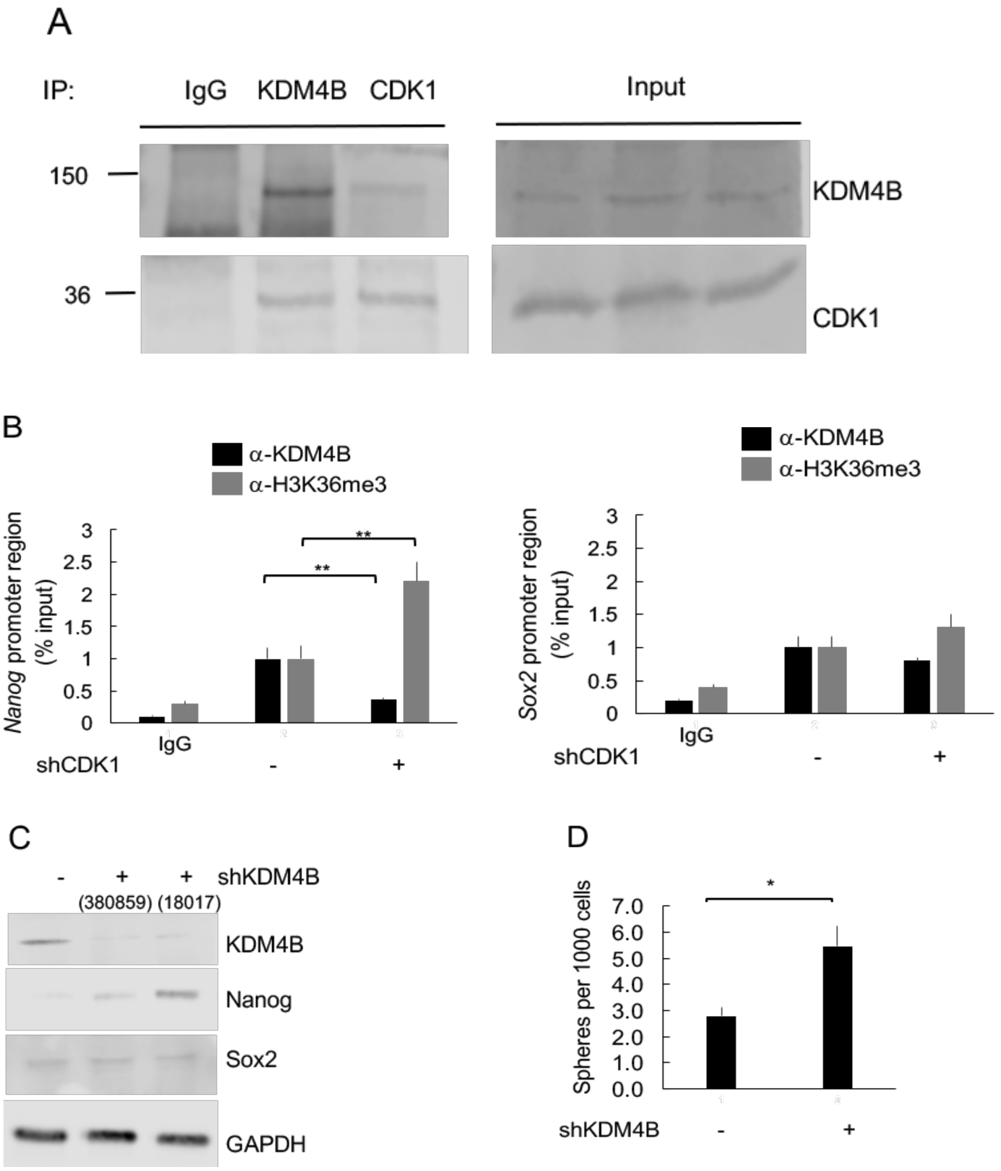

A
IP:
IgG
KDM4B
CDK1
Input
150
36
KDM4B
CDK1
B
α-KDM4B
α-H3K36me3
Nanog promoter region (% input)
3
2.5
2
1.5
1
0.5
0
**
**
IgG
shCDK1
-
+
α-KDM4B
α-H3K36me3
Sox2 promoter region (% input)
3
2.5
2
1.5
1
0.5
0
IgG
shCDK1
-
+
C
-
+
+
shKDM4B
(380859)
(18017)
KDM4B
Nanog
Sox2
GAPDH
D
Spheres per 1000 cells
7.0
6.0
5.0
4.0
3.0
2.0
1.0
0.0
*
shKDM4B
-
+

Supplementary Table S1.

Primers for Chromatin-IP	
SOX2 promoter	Fw. GGT ACC GGC CAA AGA GCT GAG TTG GA<br>Rev. AAG CTT GAG GAC AAC TGG AAT CAG GAT C
NANOG promoter	Fw. GTT CTG TTG CTC GGT TTT CT<br>Rev. TCC CGT CTA CCA GTC TCA CC
Primers for RT-PCR	
KDM5B	Fw. CAT CAC TGG CAT GTT GTT CAA ATT C<br>Rev. GAA TGT AGT AAG CCA CAA GAA GC
CDK1	Fw. CCT AGT ACT GCA ATT CGG GAA ATT<br>Rev. CCT GGA ATC CTG CAT AAG CAC
SOX2	Fw. AGC TCG CAG ACC TAC ATG AA<br>Rev. CCG GGG AGA TAC ATG CTG AT
NANOG	Fw. ACC CAG CTG TGT GTA CTC AA<br>Rev. GGA AGA GTA AAG GCT GGG GT
GAPDH	Fw. GTC ATC ATC TCT GCC CCC TCT GCT<br>Rev. CTT CTT GAT GTC ATC ATA TTT G
Primers for Subcloning	
KDM5B^{S1384A}	Fw. GGG TCT CAC TGG TGC GCT TCG GTC AGT CT<br>Rev. AGA CTG ACC GAA GCG CAC CAG TGA GAC CC
GST-KDM5B^{S1456A}	Fw. GCA GAA CGA ACT AAT TCA TAG GCA CGC TCT CTC TCT AAC TTG AA<br>Rev. TTC AAG TTA GAG AGA GAG CGT GCC TAT GAA TTA GTT CGT TCT GC
GST-KDM5B^{WT}	Fw. CGC GGA TCC ATG CAG TCT CTC AGA<br>Rev. CCG CTC GAG TTA CTT TCG GCT TGG T

Figure1

A

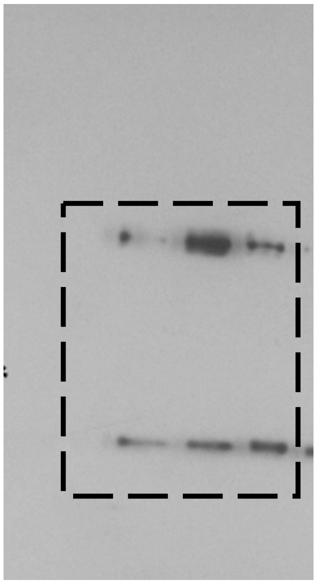

B

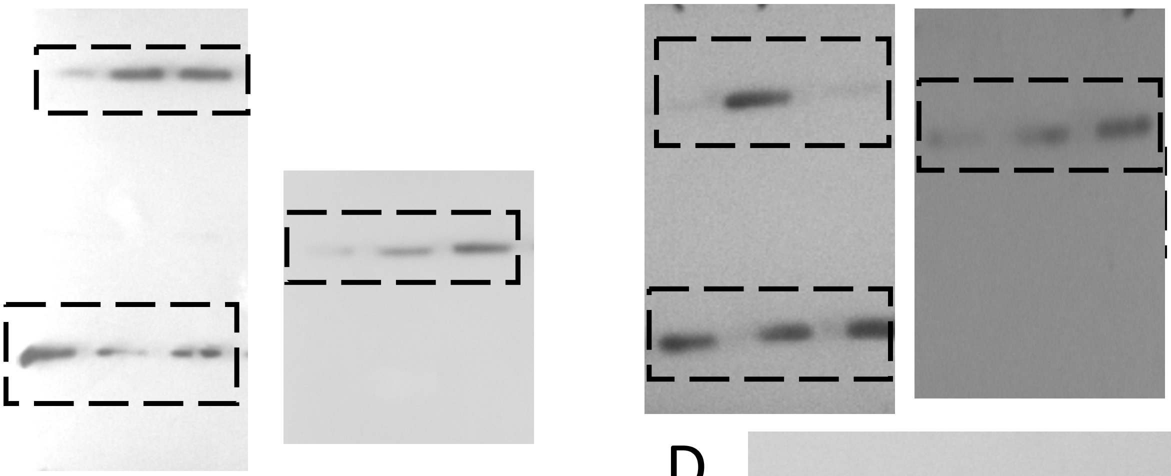

C

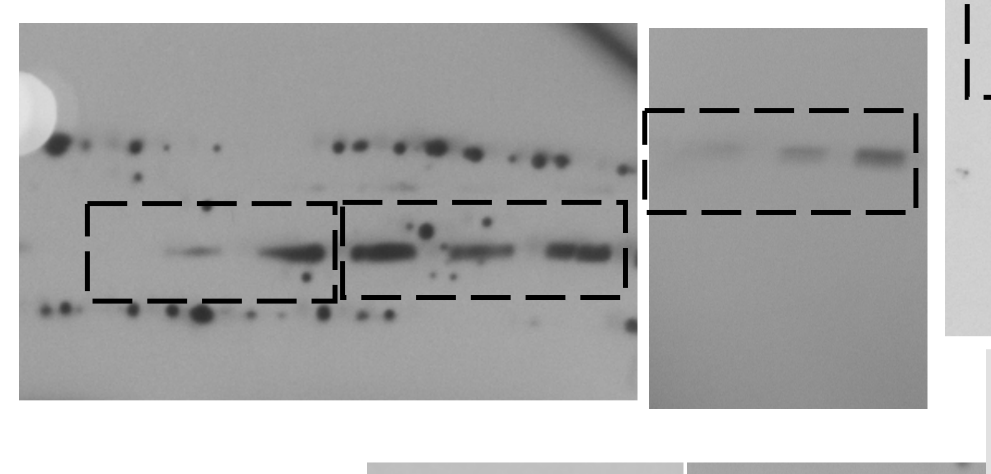

D

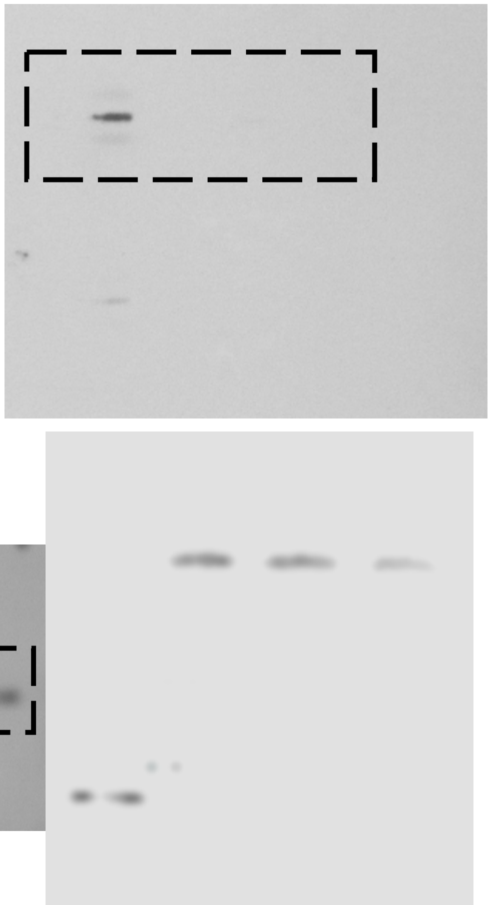

Figure 2

C

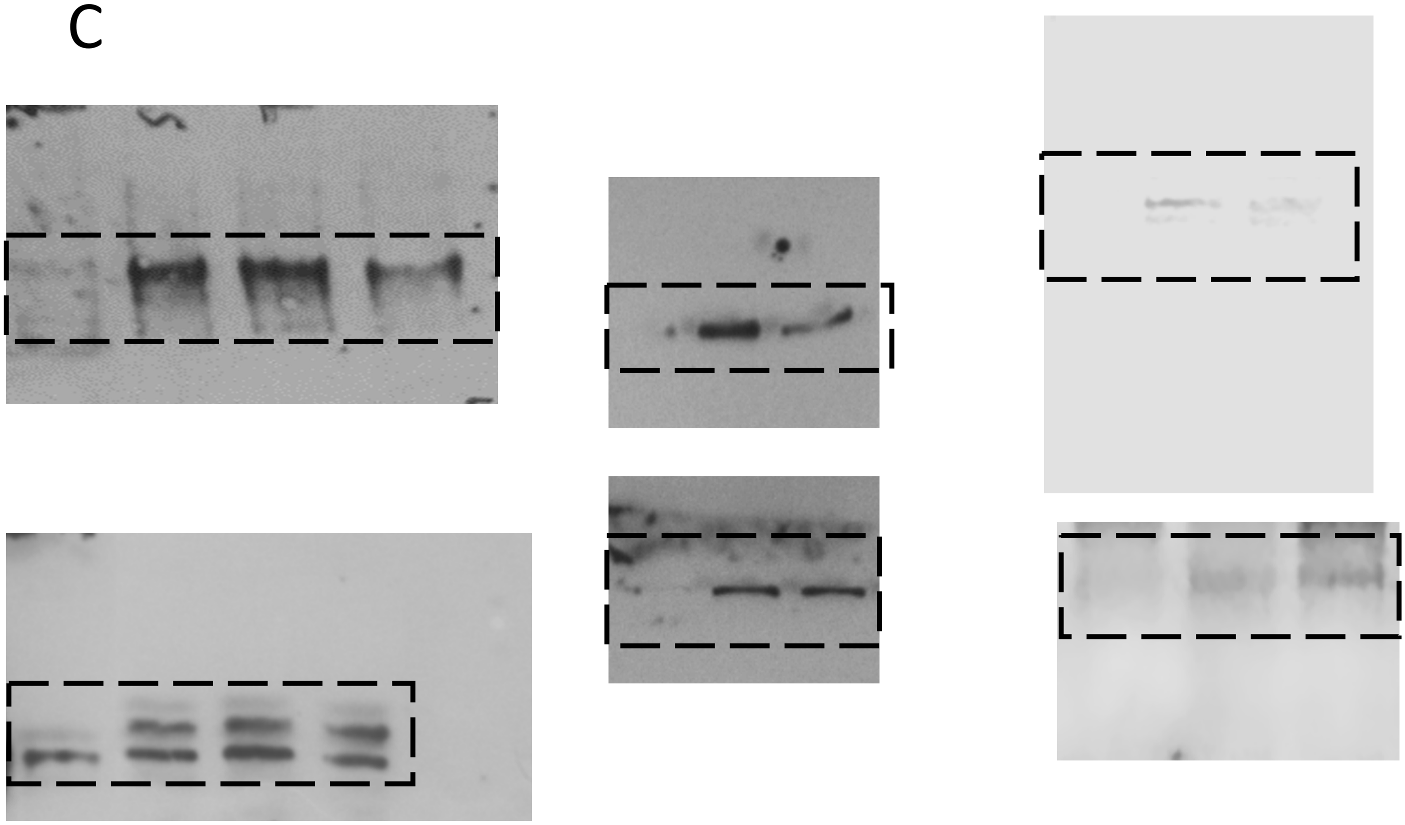

Figure 3A

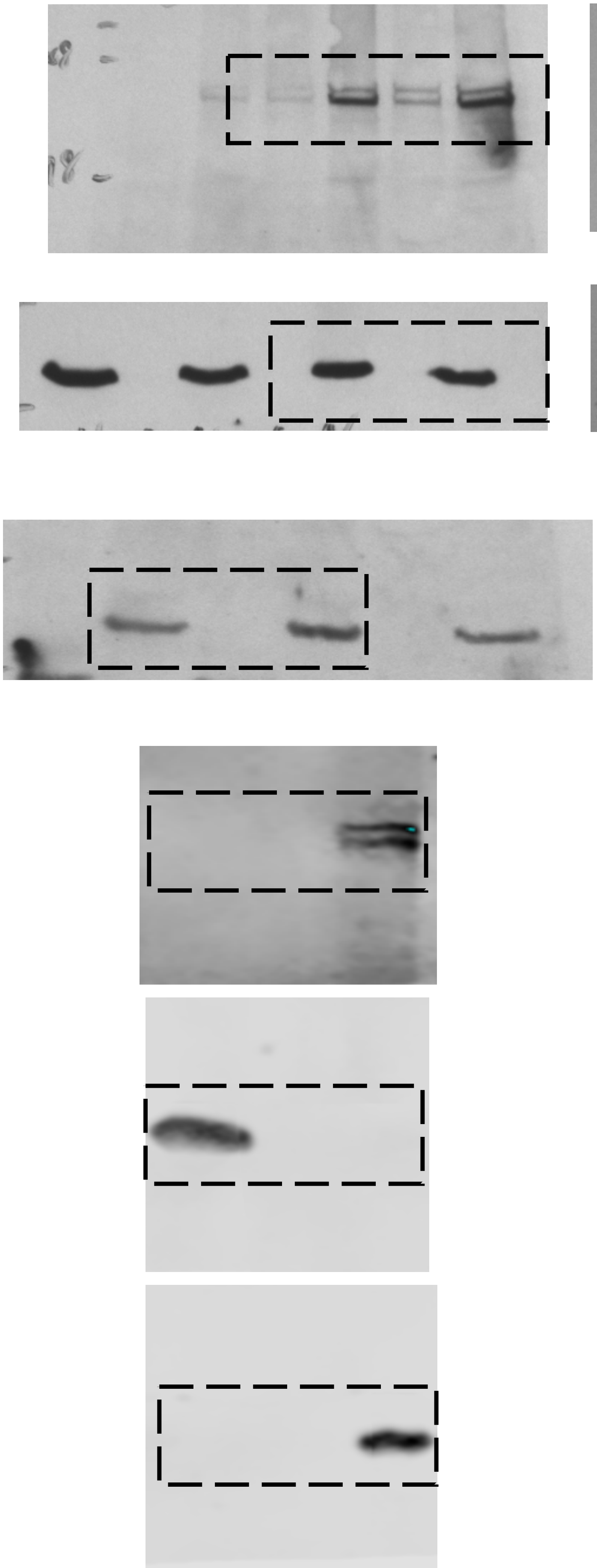

Figure 3B

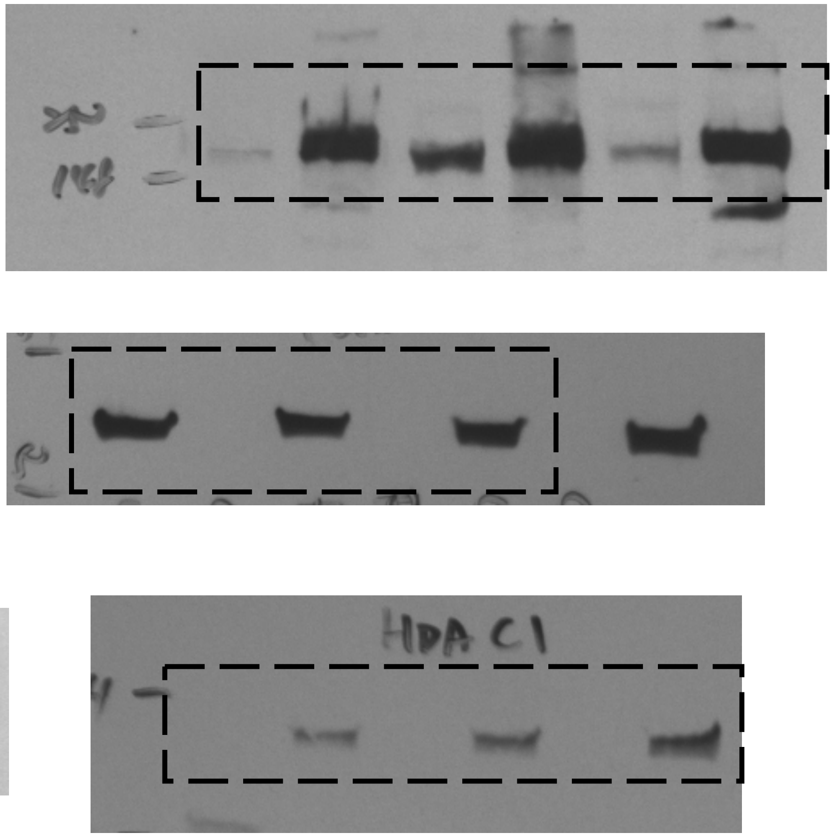

Figure 4D

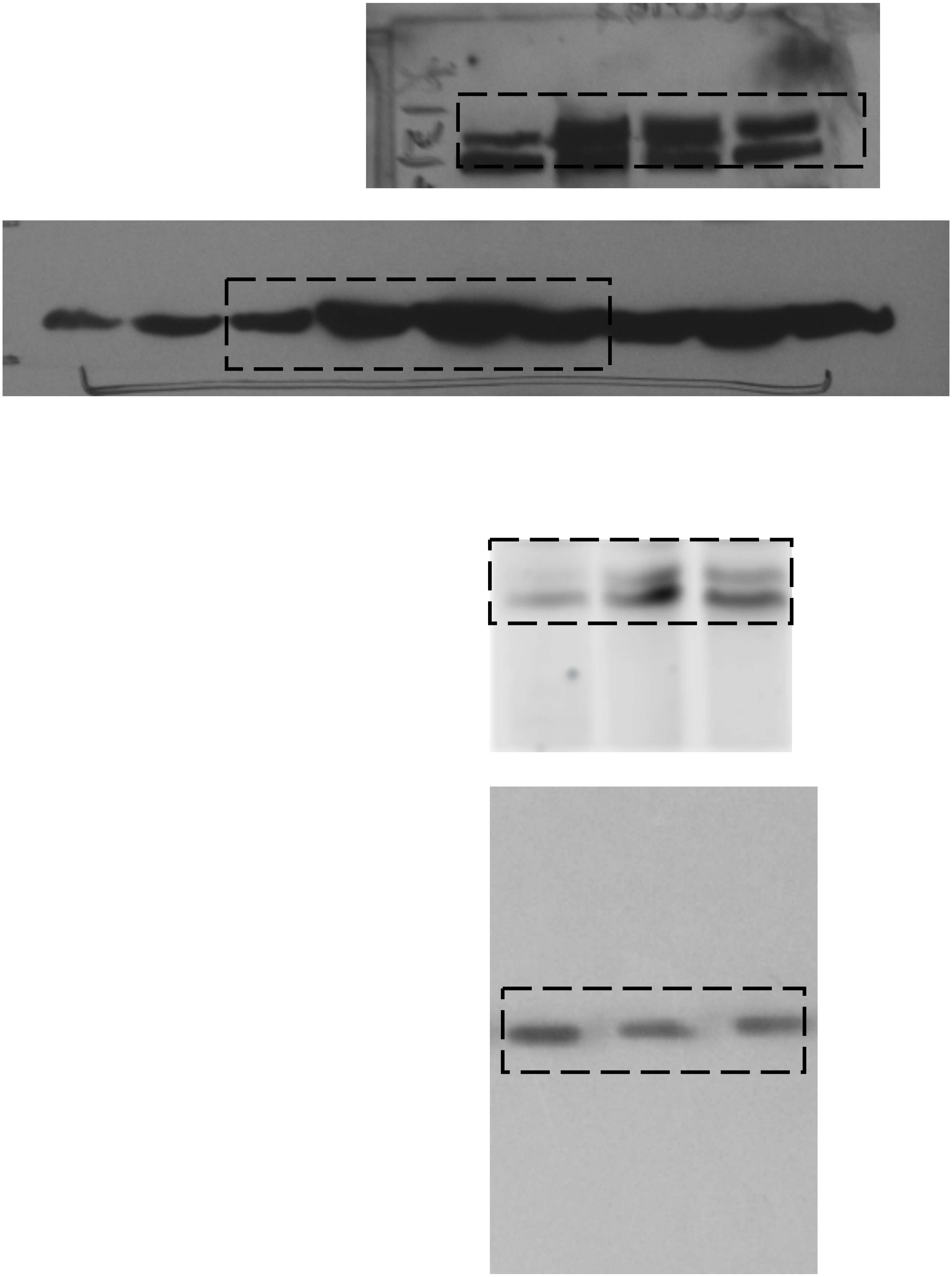

Figure 5B

MDA-MB-231 +/- shKDM5B or FLAG-KDM5B

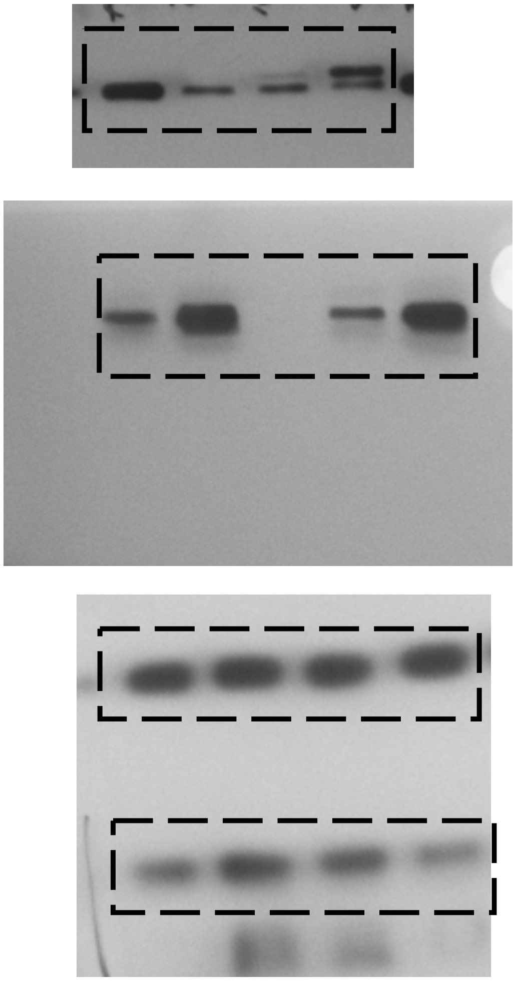

MDA-MB-231 +/- shKDM5B

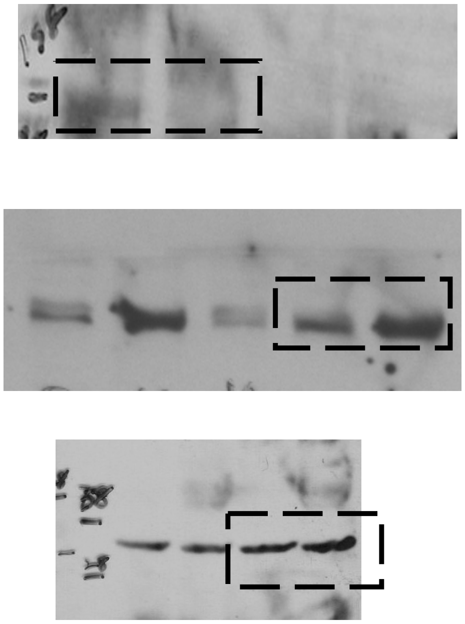

MDA-MB-231 +/- shKDM5B

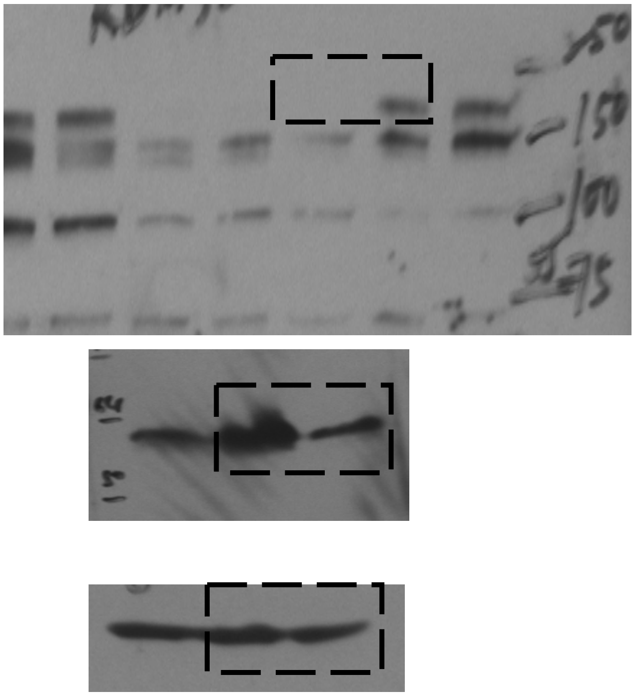

Figure 5C

MDA-MB-453+/- flKDM5B or shKDM5B

MDA-MB-231 +/- shCDK1

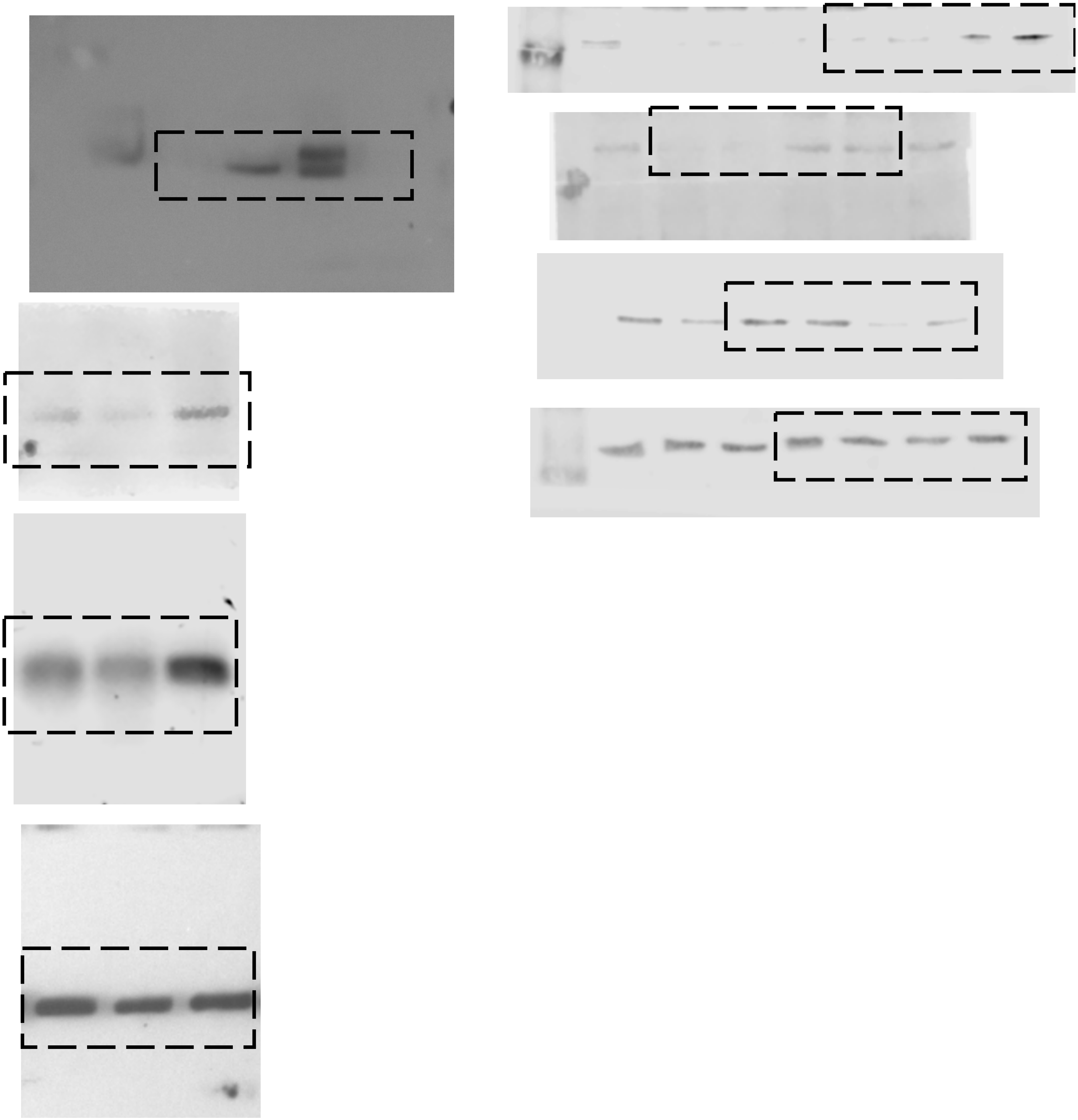

Figure 5C

HCC70 +/- shKDM5B

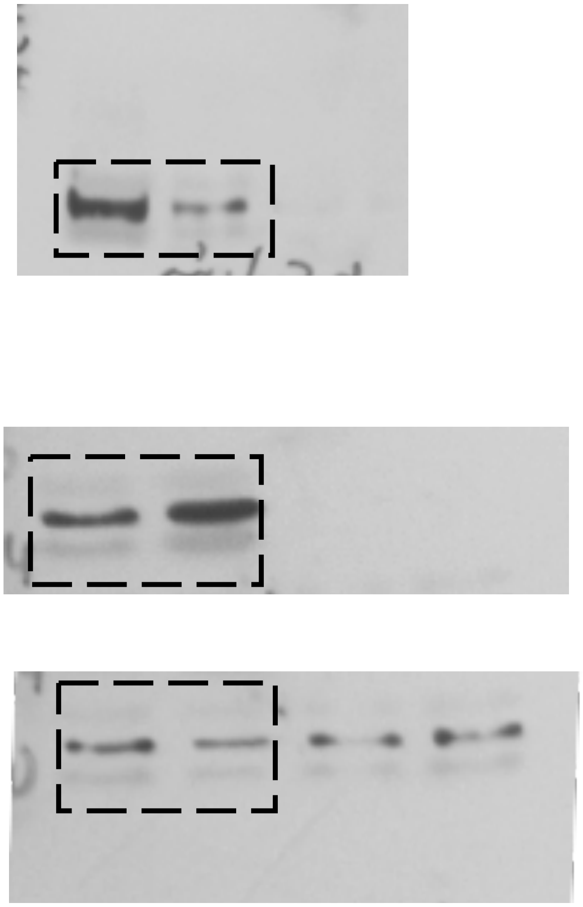

HCC1937 +/- shKDM5B

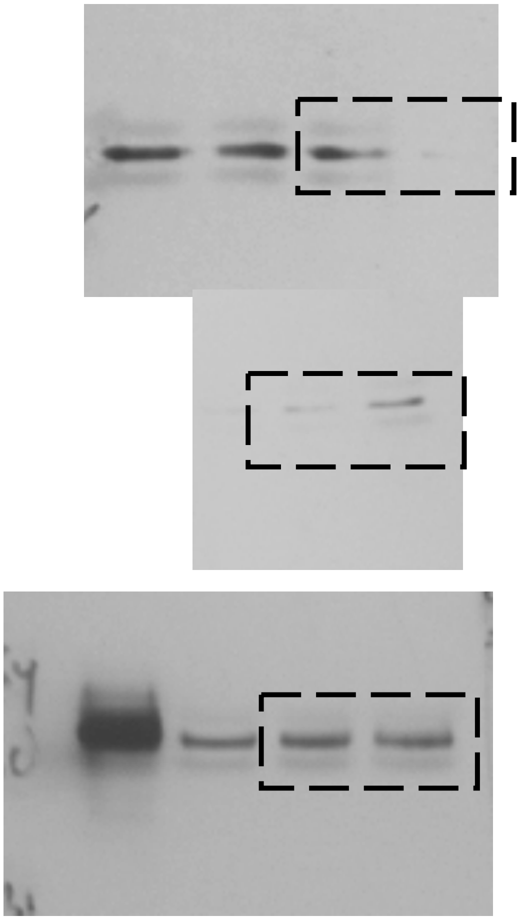

MDA-MB-231

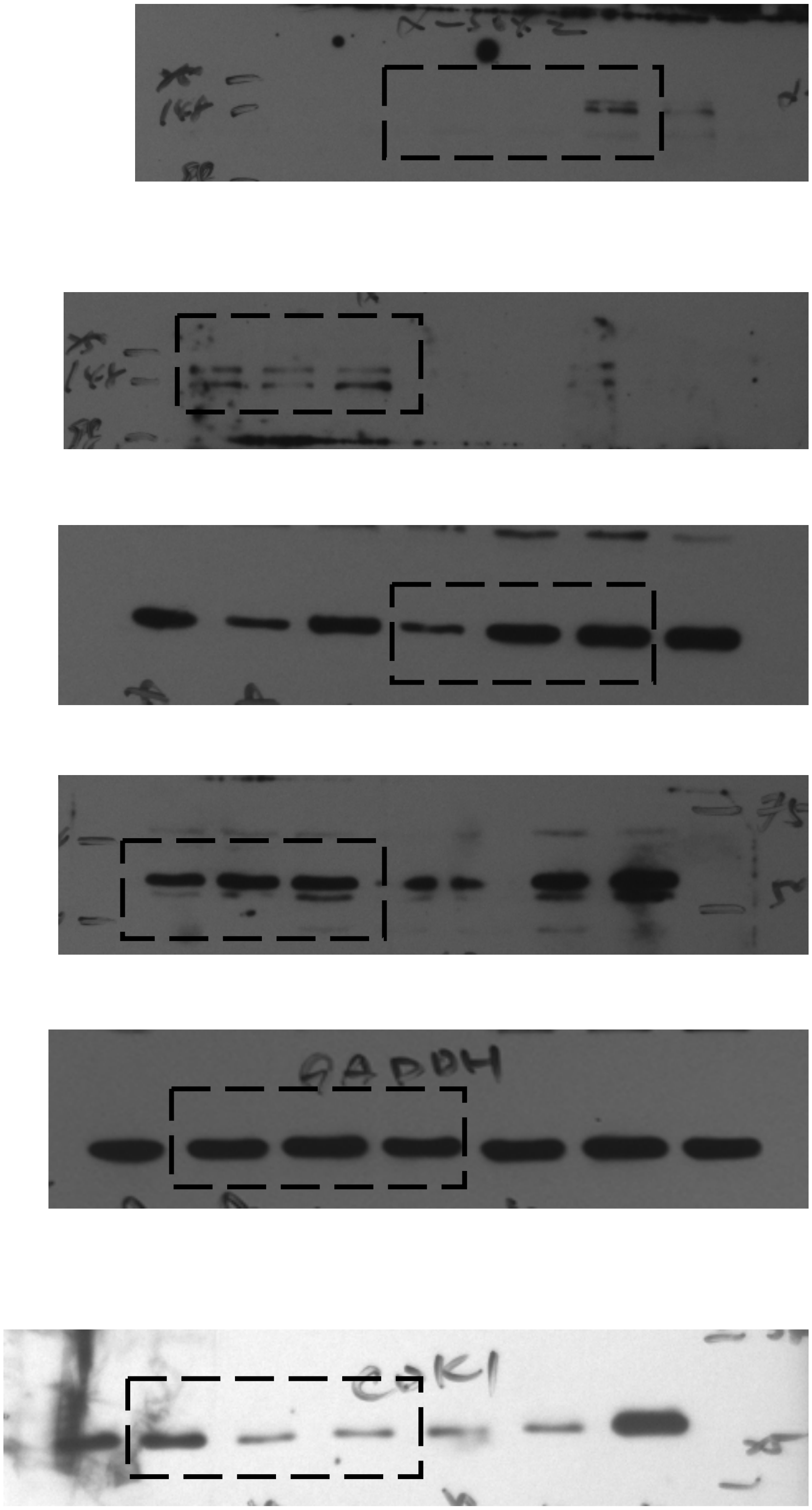

Figure 5D

Upper panel

lower panel

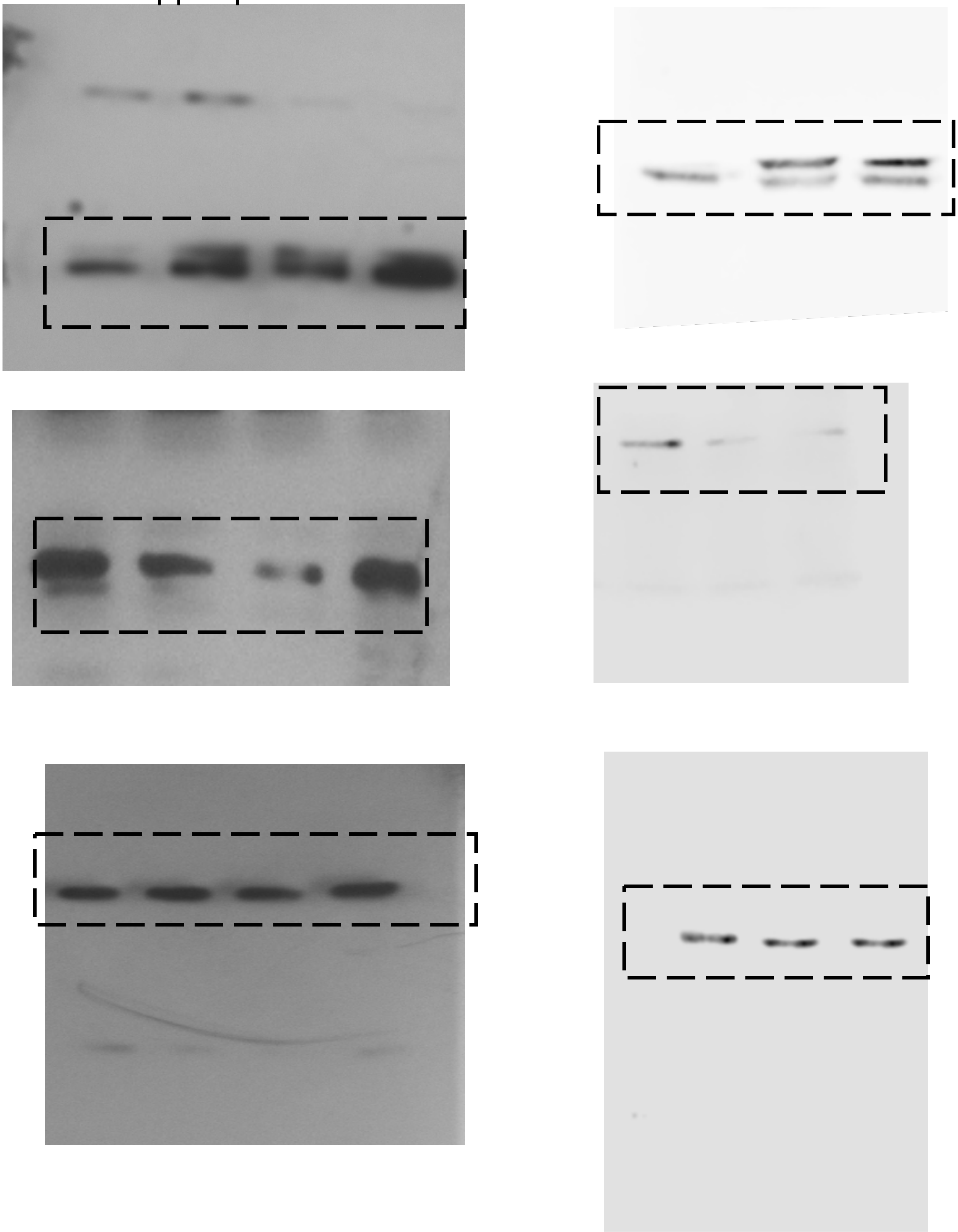

Figure 6A

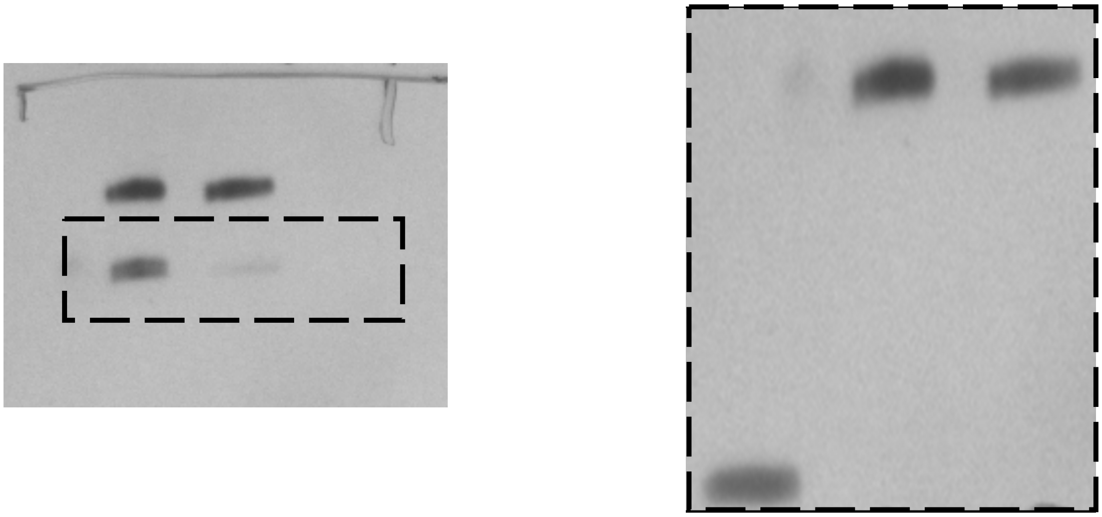

Figure 6B

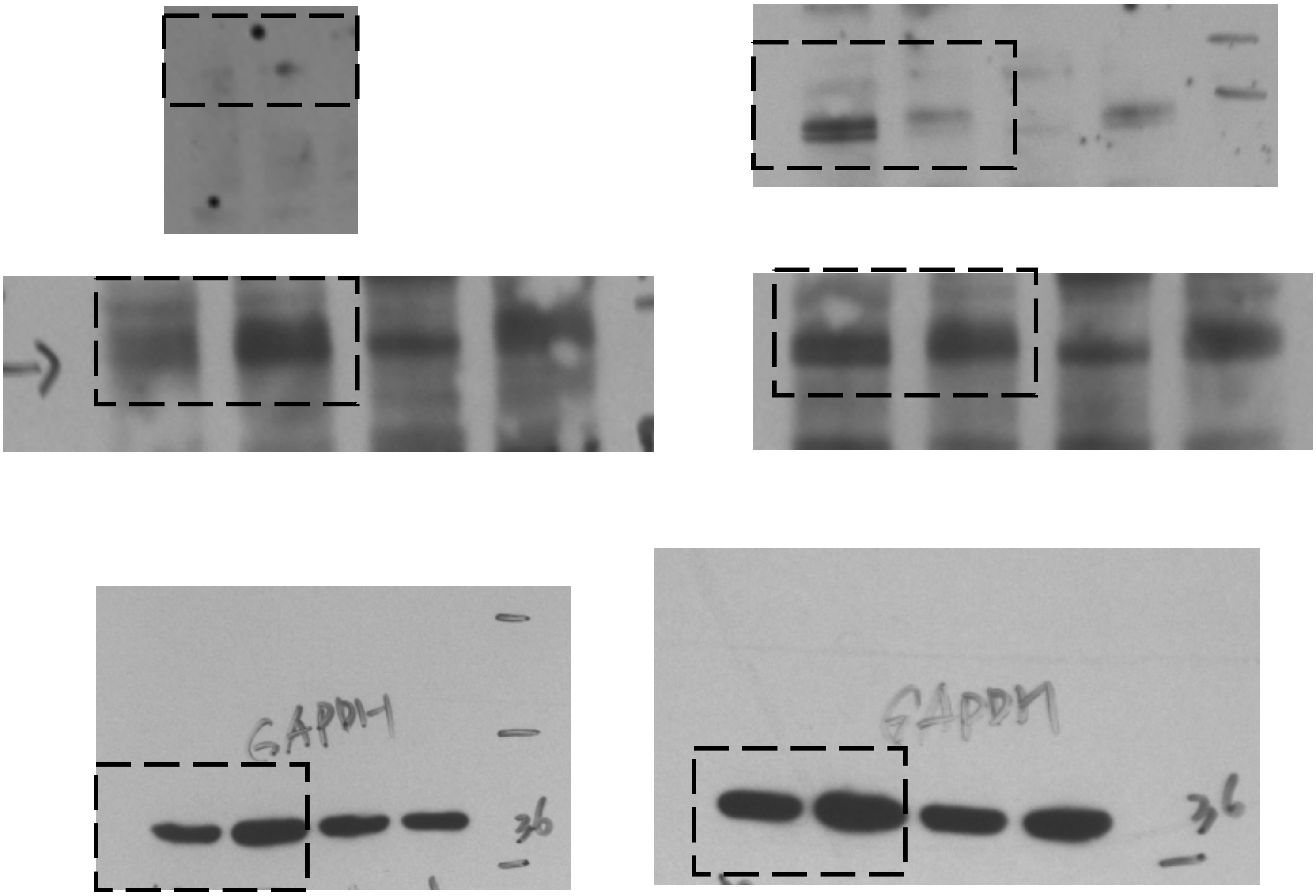

Figure 6C

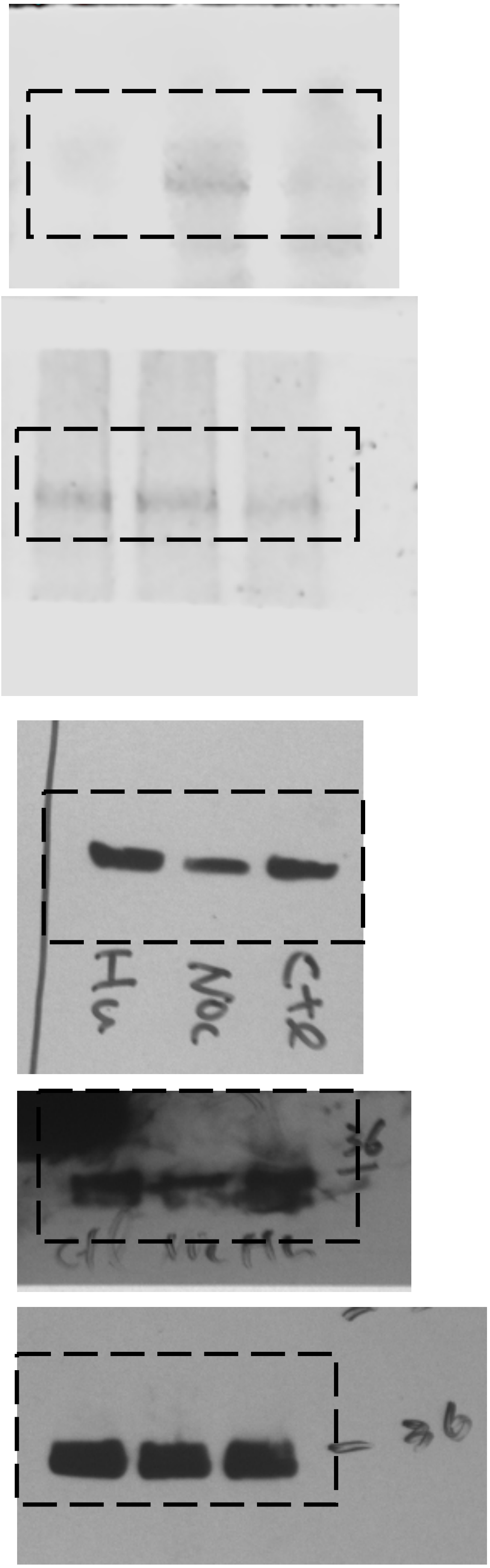

Figure 8A

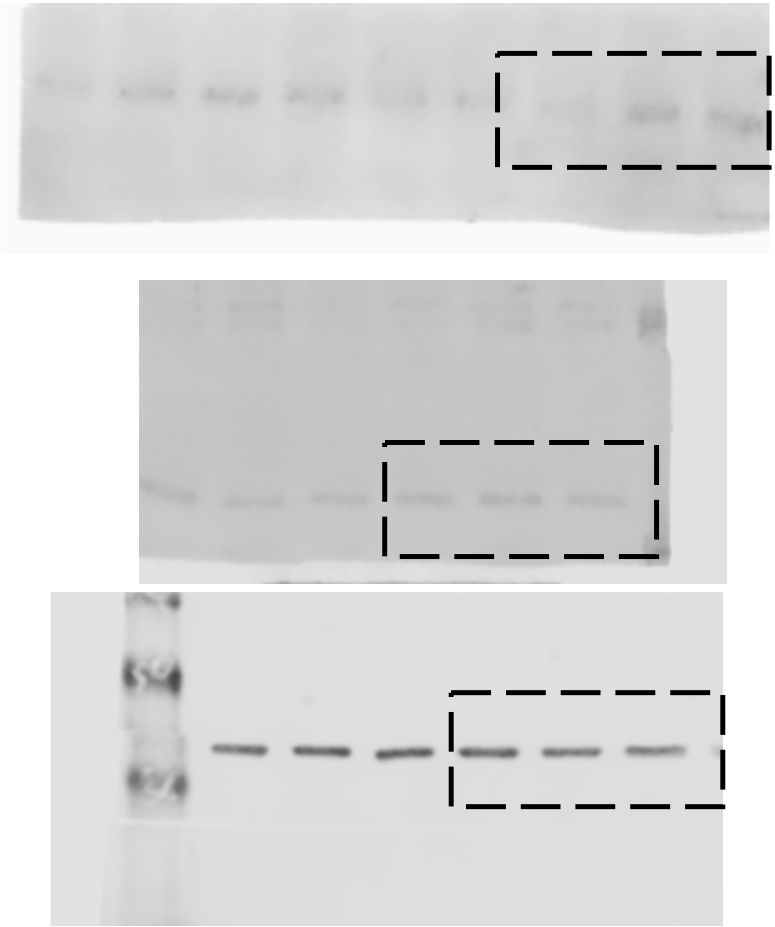

Figure 8B

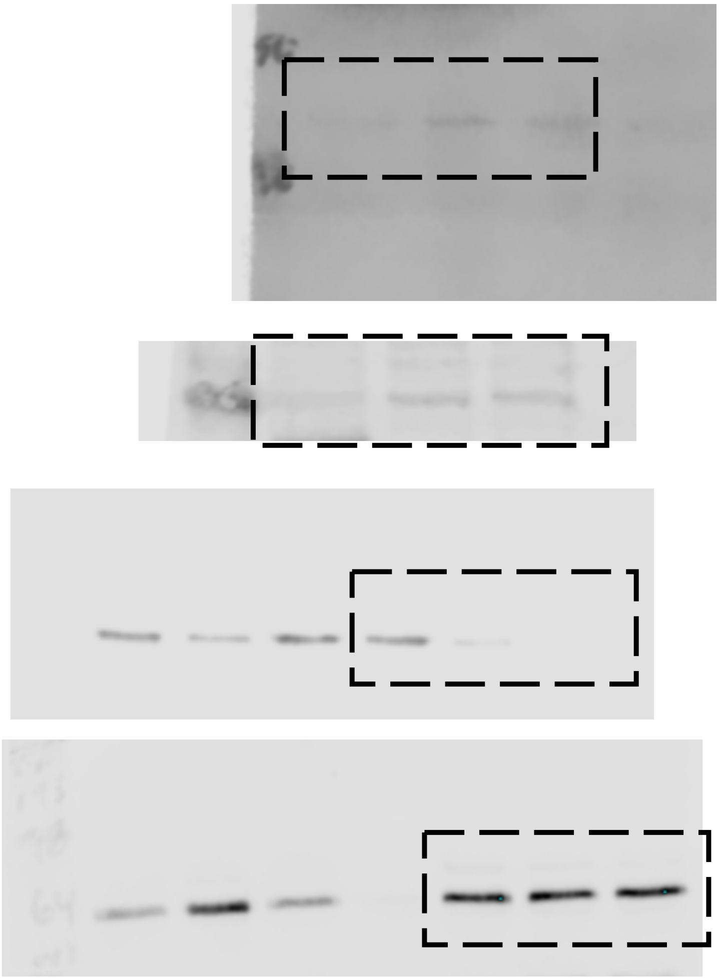

Figure S1

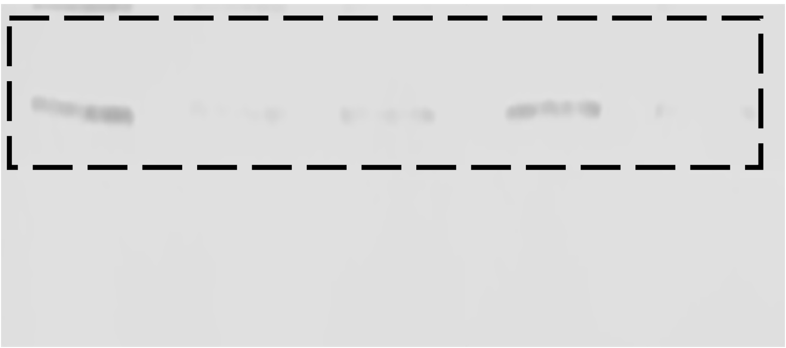

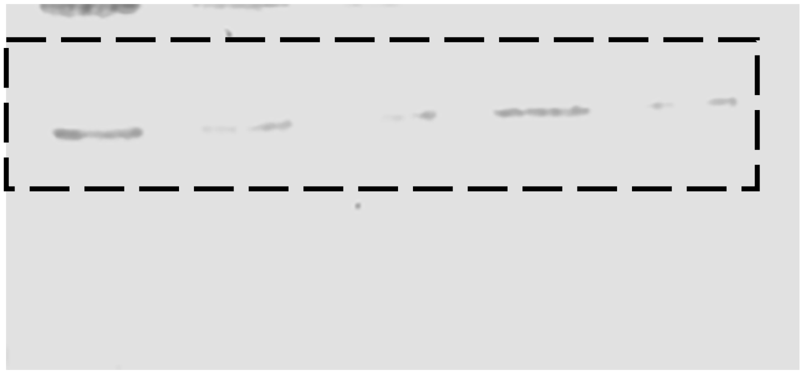

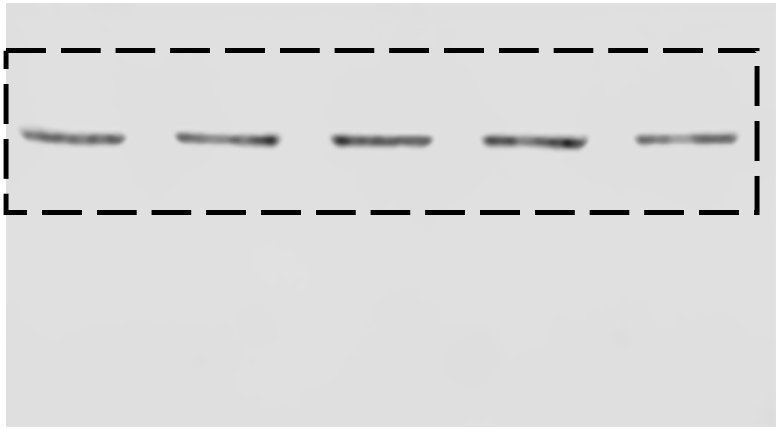

Figure S5A

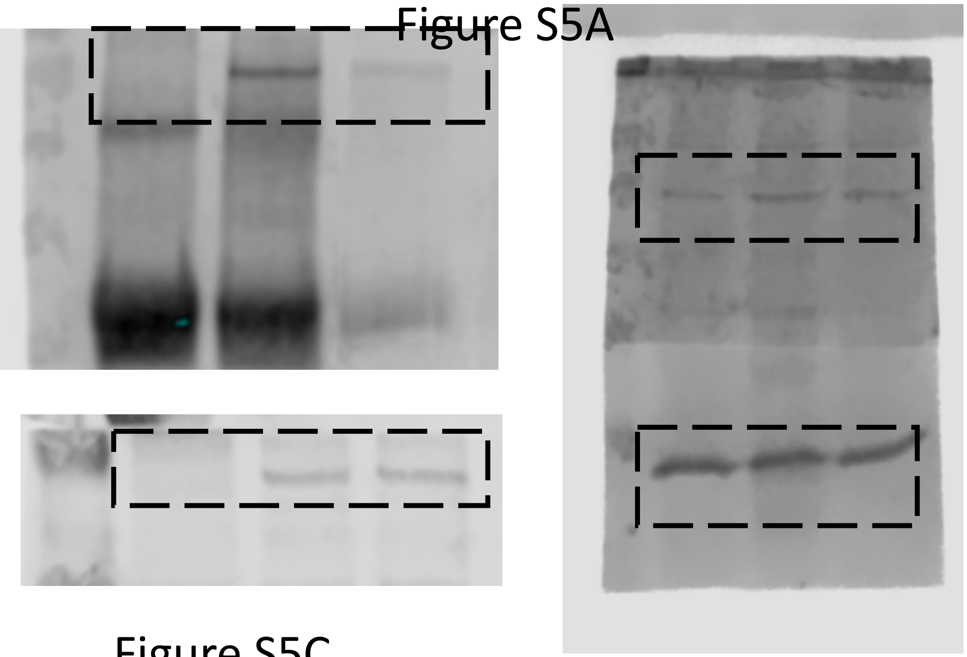

Figure S5C

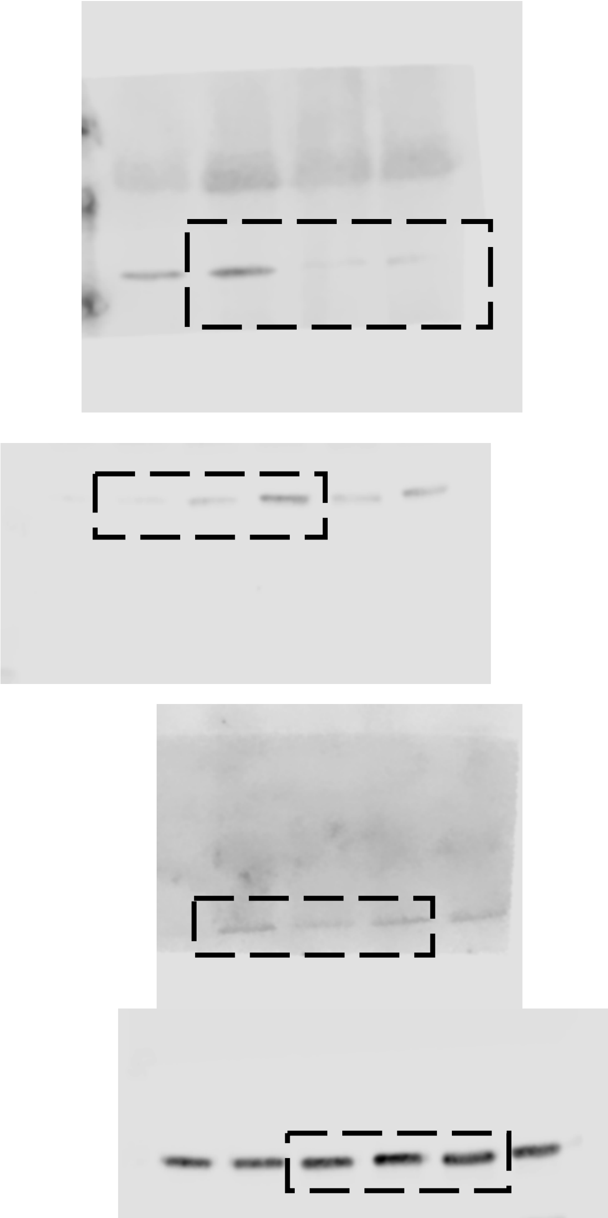

Supplement: Supplementary file 1 — Supplementary info [file 41598_2019_54184_MOESM1_ESM.pdf]
